# Immune Responses to Broad-Spectrum Antibiotic Treatment and Fecal Microbiota Transplantation in Mice

**DOI:** 10.3389/fimmu.2017.00397

**Published:** 2017-04-19

**Authors:** Ira Ekmekciu, Eliane von Klitzing, Ulrike Fiebiger, Ulrike Escher, Christian Neumann, Petra Bacher, Alexander Scheffold, Anja A. Kühl, Stefan Bereswill, Markus M. Heimesaat

**Affiliations:** ^1^Department of Microbiology and Hygiene, Charité – University Medicine Berlin, Berlin, Germany; ^2^Department of Cellular Immunology, Clinic for Rheumatology and Clinical Immunology, Charité – University Medicine Berlin, Berlin, Germany; ^3^German Rheumatism Research Center (DRFZ), Leibniz Association, Berlin, Germany; ^4^Department of Medicine I for Gastroenterology, Infectious Diseases and Rheumatology, Research Center ImmunoSciences (RCIS), Charité – University Medicine Berlin, Berlin, Germany

**Keywords:** microbiota, antibiotics, secondary abiotic (gnotobiotic) mice, fecal microbiota transplantation, innate and adaptive immunity, bacterial recolonization, mucosal and systemic immune responses

## Abstract

Compelling evidence demonstrates the pivotal role of the commensal intestinal microbiota in host physiology and the detrimental effects of its perturbations following antibiotic treatment. Aim of this study was to investigate the impact of antibiotics induced depletion and subsequent restoration of the intestinal microbiota composition on the murine mucosal and systemic immunity. To address this, conventional C57BL/6j mice were subjected to broad-spectrum antibiotic treatment for 8 weeks. Restoration of the intestinal microbiota by peroral fecal microbiota transplantation (FMT) led to reestablishment of small intestinal CD4^+^, CD8^+^, and B220^+^ as well as of colonic CD4^+^ cell numbers as early as 7 days post-FMT. However, at d28 following FMT, colonic CD4^+^ and B220^+^ cell numbers were comparable to those in secondary abiotic (ABx) mice. Remarkably, CD8^+^ cell numbers were reduced in the colon upon antibiotic treatment, and FMT was not sufficient to restore this immune cell subset. Furthermore, absence of gut microbial stimuli resulted in decreased percentages of memory/effector T cells, regulatory T cells, and activated dendritic cells in the small intestine, colon, mesenteric lymph nodes (MLN), and spleen. Concurrent antibiotic treatment caused decreased cytokine production (IFN-γ, IL-17, IL-22, and IL-10) of CD4^+^ cells in respective compartments. These effects were, however, completely restored upon FMT. In summary, broad-spectrum antibiotic treatment resulted in profound local (i.e., small and large intestinal), peripheral (i.e., MLN), and systemic (i.e., splenic) changes in the immune cell repertoire that could, at least in part, be restored upon FMT. Further studies need to unravel the distinct molecular mechanisms underlying microbiota-driven changes in immune homeostasis subsequently providing novel therapeutic or even preventive approaches in human immunopathologies.

## Introduction

The human gastrointestinal tract harbors a plethora of microorganisms, including bacteria, viruses, fungi, helminths, and protozoa that are referred to as commensal microbiota. Bacteria, however, constitute the vast majority of the intestinal microbiota (1). A recent study revealed that the ratio of human cells and bacteria is close to 1:1 with absolute numbers approximating 10^13^ each (2). Hence, a more detailed examination of distinct host-microbiota interactions remains of utmost interest. Under steady-state conditions, this interaction is largely defined by mutual benefits. The host provides the microbiota with a physiological niche in a nutrient rich environment, while the microbiota exerts various beneficial functions for the host such as vitamin production (3), digestion of dietary compounds (4), and protection from pathogens (5).

One important aspect underlining the indispensability of gut microorganisms is their contribution to the development, maturation, and regulation of the immune system (6). Studies in isolator-raised germfree (GF) mice revealed fundamental impairments regarding the development and differentiation of immune compartments including Peyer’s patches (PP) and mesenteric lymph nodes (MLN) as indicated by decreased IgA levels, and cellular defects of intestinal epithelial and lamina propria (LP) lymphocytes (7). Given that the alteration of the intestinal microbiota composition, termed dysbiosis, is associated with defined immunopathological conditions including inflammatory bowel diseases (8, 9), allergies (10), type 2 diabetes mellitus (11), obesity (12), anxiety, depression (13), and autism (14), the orchestrated interplay between the commensal microbiota and host cells plays a pivotal role in maintaining immune homeostasis and host cell physiology.

This rationale is further supported by compelling evidence derived from studies with defined bacterial strains that induce the development and expansion of distinct immune cell subsets. For instance, segmented filamentous bacteria have been identified as potent inducers of the IL-17-producing T helper (Th) 17 cells (15), while *Clostridium* species of clusters IV and XIVa promoted accumulation of regulatory T cells (Treg) in the colonic LP of mice (16).

Antibiotic treatment, besides being one of the greatest achievements in the history of medicine, results in disruption of intestinal microbial communities as collateral damage with long-term consequences after cessation of therapy (17). Many antibiotic compounds have been shown to render the host susceptible to infection by several pathogens including *Salmonella* species (18), vancomycin-resistant *Enterococcus* spp. (19), and *Clostridium difficile* (20). *C. difficile* toxin-induced enterocolitis, for instance, represents one of the biggest antibiotics-related health-care problems with potentially fatal outcome (20). Remarkably, even short-term application of antimicrobial compounds such as clindamycin induces long-lasting decreases in enteric microbial diversity and renders mice susceptible to *C. difficile* colonization and infection (17).

Furthermore, there is profound evidence regarding the impact of antibiotic treatment on immune cell homeostasis. For instance, mice treated with vancomycin or colistin from birth on displayed decreased numbers of isolated lymphoid follicles, a tertiary lymphoid tissue, in the small and large intestines (21). Moreover, treatment of mice with an antibiotic cocktail consisting of neomycin, vancomycin, and metronidazole resulted in lower intestinal expression of regenerating islet-derived protein 3 gamma, an antimicrobial peptide directed against Gram-positive bacteria (22), whereas treatment with vancomycin resulted in reduced Treg numbers in the colon (16). Reduction of the Treg population could also be observed in the murine MLN and PP upon microbiota depletion by broad-spectrum antibiotic treatment (23). Whether the observed effects on the immune system following antimicrobial treatment were rather primarily due to the alterations of microbial communities and/or distinct compound-related mechanisms, however, remains unanswered.

In this study, we therefore aimed to further elucidate the interplay of the triangle relationship between intestinal microbiota, antibiotics and the immune system in more detail. To address this, we performed a comprehensive survey of distinct immune cell subsets, including CD4^+^ and CD8^+^ T lymphocytes, B lymphocytes, memory T cells, activated dendritic cells (DC), and Treg in intestinal and systemic compartments of mice that were virtually depleted of microbiota through broad-spectrum antibiotic treatment as compared to secondary abiotic (ABx) mice following fecal microbiota transplantation (FMT) and to conventionally colonized mice. Moreover, we analyzed both pro- and anti-inflammatory cytokines including IFN-γ, IL-17, IL-22, and IL-10 expressed by CD4^+^ lymphocytes following broad-spectrum antibiotic treatment and FMT.

## Materials and Methods

### Mice

All animals were bred, raised, and housed in the facilities of the “Forschungseinrichtungen für Experimentelle Medizin” (Charité – University Medicine Berlin, Germany) under specific-pathogen-free (SPF) conditions. Female age-matched C57BL/6j wild-type mice were used.

### Generation of Secondary Abiotic (Gnotobiotic) Mice and Reconstitution of the Intestinal Commensal Microbiota by Fecal Transplantation

In order to virtually deplete the intestinal microbiota, 8–10 weeks old mice were transferred to sterile cages and subjected to quintuple antibiotic treatment for 8 weeks as previously described (24). Three days prior to peroral FMT, the antibiotic cocktail was withdrawn and replaced by sterile drinking water. Successful eradication of the cultivable intestinal microbiota was confirmed as described previously (24). Fresh murine fecal samples were collected from 10 age- and sex-matched SPF control mice, pooled, dissolved in 10 ml sterile phosphate-buffered saline (PBS; Gibco, Life Technologies, Paisley, UK) and the supernatant perorally applied by gavage (in 0.3 ml PBS) in order to reconstitute secondary abiotic (i.e., gnotobiotic) mice with a complex intestinal microbiota.

### Sampling Procedures

Mice were sacrificed by isoflurane treatment (Abbott, Greifswald, Germany) at day 7 or day 28 post-FMT. Luminal large intestinal samples as well as *ex vivo* biopsies from spleen, MLN, ileum, and colon were taken under sterile conditions. Ileal and colonic *ex vivo* biopsies were collected from each mouse in parallel for immunological, microbiological, and immunohistochemical analysis. For immunohistochemical stainings, ileum and colon samples were immediately fixed in 5% formalin and embedded in paraffin, and sections (5 µm) were stained with distinct antibodies as described below.

### Immunohistochemistry

*In* situ immunohistochemical analysis of ileal and colonic paraffin sections was performed as previously described (25–28). Primary antibodies against cleaved caspase-3 (Asp175, Cell Signaling, Beverly, MA, USA, 1:200), Ki67 (TEC3, Dako, Glostrup, Denmark, 1:100), CD3 (#N1580, Dako, 1:10), Foxp3 (FJK-16s, eBioscience, San Diego, CA, USA, 1:100), B220 (eBioscience, 1:200), and F4/80 (# 14-4801, clone BM8, eBioscience, 1:50) were used. For each animal, the average number of positively stained cells within at least six high power fields (HPF, 400× magnification) was determined microscopically by an independent blinded investigator.

### Lymphocyte Isolation from Spleen and MLN

Single cell suspensions were generated from spleens and MLN, and erythrocytes were removed from splenic samples by 1.66% ammonium chloride. All samples were resuspended in defined volumes of PBS/0.5% bovine serum albumin (BSA) and subjected to further processing (23).

### LP Lymphocyte Isolation

Segments of the murine gut were removed and freed from fat, connective tissue, and PP, cut longitudinally, and cleared from luminal content and mucus with ice-cold PBS. The isolation of lamina propria lymphocytes (LPL) followed a standard protocol with minor modifications (29). Briefly, the intestines were cut into 0.5 cm pieces and incubated twice with 25 ml Hanks’ balanced salt solution (HBSS; Gibco) containing 1 mM dithioerythritol (Carl Roth) for 20 min at 37°C and 220 rpm. Afterward, the intestines were introduced to HBSS containing 1.3 mM ethylenediaminetetraacetic acid (Life Technologies, Eugene, OR, USA). Subsequently the cells were placed in digestion solution, containing 0.5 mg/ml collagenase A (Roche, Mannheim, Germany), 0.5 mg/ml DNAse I (Roche), 10% fetal calf serum (FCS), and 1 mM of each CaCl_2_ and MgCl_2_ (both Carl Roth). Digestion was performed through incubation for 45 min at 37°C and 220 rpm. After the incubation, the digested tissues were washed with RPMI containing 5% FCS, resuspended in 5 ml 44% Percoll (GE Healthcare, Uppsala Sweden), and overlaid on 5 ml 67% Percoll in a 15 ml Falcon tube. Percoll gradient separation was performed by centrifugation at 600 *g* for 20 min at room temperature. LPL were collected from the interphase, washed once, and suspended in PBS/0.5% BSA.

### Surface and Intracellular Stainings and Flow Cytometry

Surface staining was performed using following antibodies: FITC-anti-CD4 (Clone RM4-5; 1:200), PerCP-anti-CD8 (Clone 53-6.7; 1:100), PacBlue-anti-B220 (Clone RA3-6B2, 1:200), APC-Cy7-anti-CD25 (Clone PC61, 1:200), PE-anti-CD44 (Clone IM7, 1:200), and APC-anti-CD86 (Clone B7-2, 1:200) (all from BD Biosciences, San Jose, CA, USA).

For intracellular staining, cells were restimulated for 5 h with 10 ng/ml phorbol myristate acetate and 1 µg/ml ionomycin, in a tissue culture incubator at 37°C (both Sigma-Aldrich). Ten micrograms per milliliter brefeldin A (Sigma-Aldrich) were added to the cell suspensions after 1 h of polyclonal restimulation. Then cells were treated with LIVE/DEAD Fixable Aqua Dead Cell Stain kit (Life Technologies) and hereafter fixed with 2% paraformaldehyde (Sigma-Aldrich) for 20 min at room temperature. Cells were stained in 0.5% saponin (Sigma-Aldrich) using following antibodies: PacBlue-anti-CD4 (Clone RM4-5; 1:400), PE-Cy7-anti-IFN-γ (Clone XMG 1.2; 1:400) (both from BD Biosciences), FITC-anti-IL17A (Clone TC11-18H10.1; 1:200, BioLegend, San Diego, CA, USA), PE-anti-IL10 (Clone JESS-16E3; 1:100), and APC-anti-IL22 (Clone IL22JOP; 1:100) (both from eBioscience). All data were acquired on a MACSQuant analyzer (Miltenyi Biotec, Bergisch Gladbach, Germany) and were analyzed with FlowJo Software v10.1 (Tree star, Ashland, OR, USA).

### Real-time PCR

Expression levels of pro- and anti-inflammatory cytokines including IFN-γ, IL-22, IL-17A, and IL-10 mRNA were determined in snap frozen ileal and colonic *ex vivo* biopsies using Light Cycler Data Analysis Software (Roche) as stated elsewhere (30). The mRNA of the housekeeping gene for hypoxanthine-phosphoribosyltransferase was used as reference; the mRNA expression levels of the individual genes were normalized to the lowest measured value and expressed as fold expression (arbitrary units) (31).

For molecular analysis of the intestinal microbiota, DNA was extracted from fecal samples as described previously (24). Briefly, DNA extracts and plasmids were quantified using Quant-iT PicoGreen reagent (Invitrogen, Paisley, UK) and adjusted to 1 ng/µl. Then, abundance of the main bacterial groups of murine intestinal microbiota was assessed by quantitative real time-PCR with group-specific 16S rRNA gene primers (Tib MolBiol, Berlin, Germany) as described previously (5, 32, 33). The number of 16S rRNA gene copies per microgram DNA of each sample was determined, and frequencies of respective bacterial groups calculated proportionally to the eubacterial (V3) amplicon.

### Statistical Analysis

Medians, means, SDs, and significance levels were determined using Mann–Whitney *U* test or one-way analysis of variance with Tukey’s post hoc test for multiple comparisons (GraphPad Prism Software v6, La Jolla, CA, USA) as indicated. Two-sided *p* values ≤ 0.05 were considered significant. Data shown were pooled from two independent experiments (*n* = 10–15 per group).

## Results

### Depletion and Reconstitution of the Murine Intestinal Microbiota following Broad-Spectrum Antibiotic Treatment and FMT

**Figure 1:**
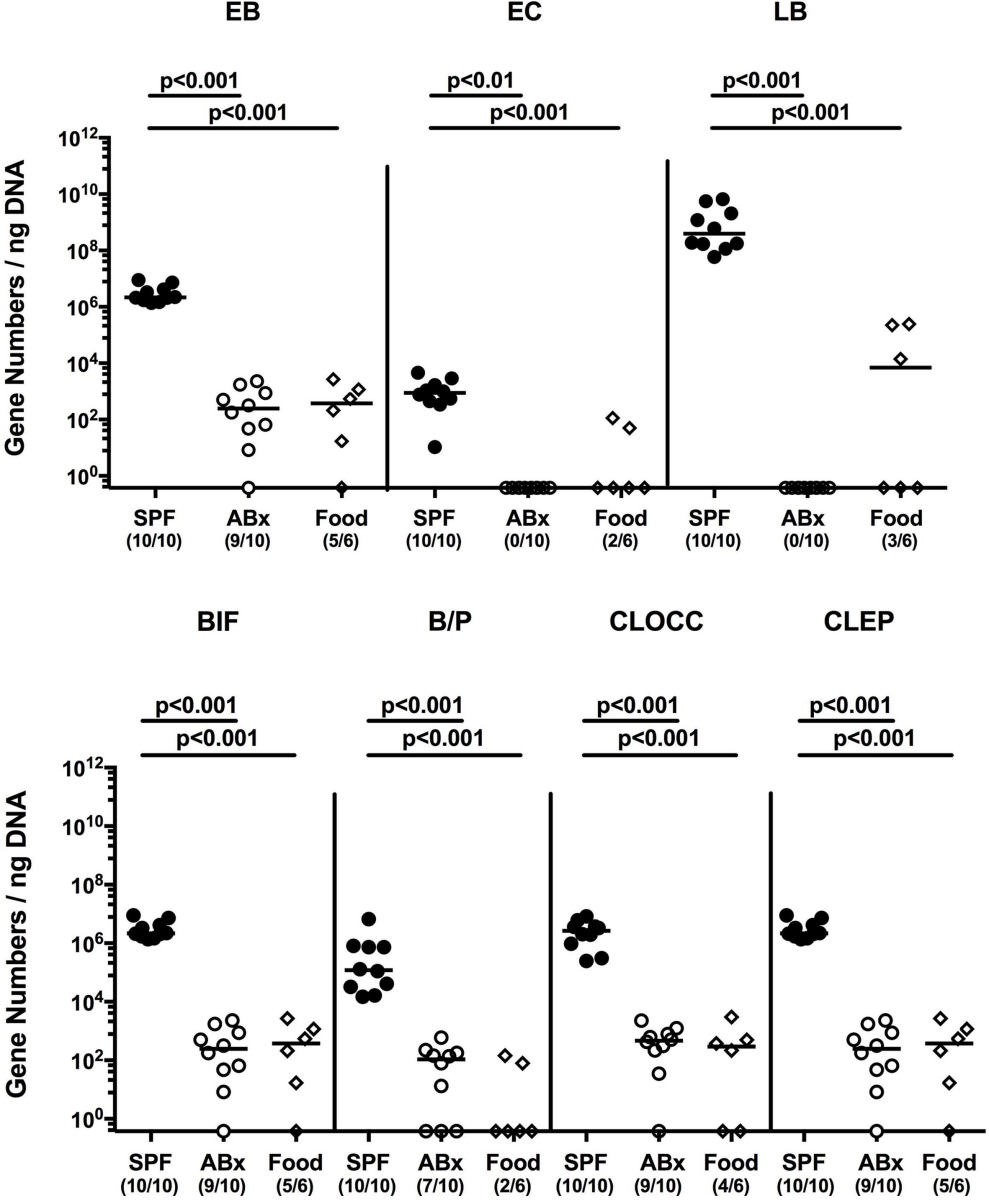
**Intestinal microbiota composition of conventional and secondary abiotic mice as compared to autoclaved food pellets**. The intestinal microbiota composition was analyzed in fecal samples derived from conventionally colonized (SPF) mice and mice subjected to an 8 weeks course of broad-spectrum antibiotic treatment [thereby generating secondary abiotic (ABx) mice] by quantitative real-time PCR amplifying variable regions of the bacterial 16S rRNA gene and compared to the bacterial composition detected in sterilized (autoclaved) food pellets. The following main intestinal bacterial groups were determined (expressed as 16S rRNA gene numbers per nanogram DNA): enterobacteria (EB), enterococci (EC), lactic acid bacteria (LB), bifidobacteria (BIF), *Bacteroides/Prevotella* spp. (BP), *Clostridium coccoides* group (CLOCC), and *Clostridium leptum* group (CLEP). Numbers of samples harboring the respective bacterial group out of the total number of analyzed samples are given in parentheses.

To confirm successful depletion of the intestinal microbiota, we applied cultural analyses of fecal samples derived from ABx mice. In fact, all fecal samples were culture negative for aerobic, microaerobic, and obligate anaerobic species as assessed by direct plating and enrichment procedures (not shown). To additionally assess abundance of fastidious and uncultivable intestinal bacteria, we next determined the main bacterial groups abundant in the murine intestinal tract by quantitative 16S rRNA-based PCR analysis of fecal samples derived from conventionally colonized and ABx mice as compared to respective bacterial groups abundant in autoclaved food pellets. In ABx mice, bacterial 16S rRNA gene numbers were decreased by up to 10 orders of magnitude as compared to conventional SPF controls (*p* < 0.001; Figure 1). Remarkably, mean 16S rRNA gene numbers in fecal samples derived from ABx mice and autoclaved food pellets were comparable, indicating a successful and biologically relevant depletion of the intestinal microbiota following broad-spectrum antibiotic treatment. Notably, one cannot differentiate whether detected 16S rRNA gene numbers in ABx mice were derived from avital (“dead”) or viable fastidious/uncultivable bacterial cells. We next determined the efficiency of intestinal microbiota reconstitution upon FMT of ABx mice. As assessed by molecular methods, 16S rRNA gene numbers of the main bacterial intestinal microbiota groups were comparable in conventional mice and ABx mice at days 7 and 28 post-FMT (Figure S1 in Supplementary Material) indicating a successful reconstitution of the intestinal microbiota by FMT.

### Macroscopic and Microscopic Sequelae of Broad-Spectrum Antibiotic Treatment and FMT

**Figure 2:**
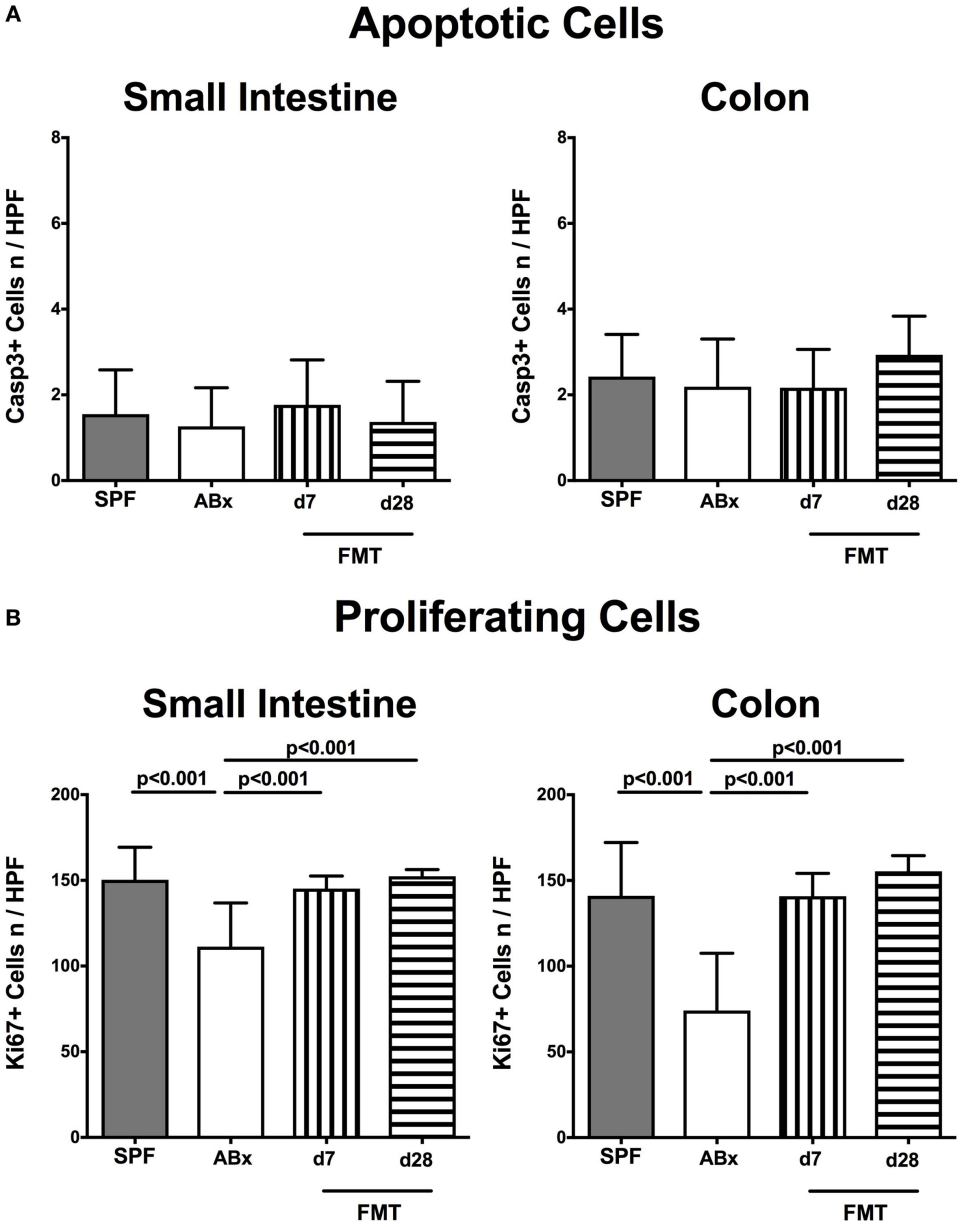
**Apoptotic and proliferating cells in small and large intestinal epithelia of secondary abiotic and microbiota-reconstituted mice**. The average numbers of **(A)** apoptotic (positive for caspase-3, Casp3) and **(B)** proliferating cells (positive for Ki67) in at least six representative high power fields (HPFs, 400× magnification) per animal were determined in immunohistochemically stained small intestinal and colonic *ex vivo* biopsies derived from naive conventional mice (SPF, gray bars), secondary abiotic mice (ABx, white bars), and abiotic mice reconstituted with murine microbiota at day (d) 7 (bars with vertical lines) and d28 (bars with horizontal lines) following fecal microbiota transplantation (FMT).

Given that neither antibiotic treatment nor FMT affected mice clinically and resulted in macroscopic sequelae such as wasting, diarrhea, or occurrence of blood in fecal samples (not shown), we assessed potential microscopic changes in intestinal *ex vivo* biopsies derived from ABx and FMT mice. To address this, we determined numbers of apoptotic cells in small and large intestinal paraffin sections following staining against caspase-3, given that apoptosis is an established parameter used for histopathological evaluation and grading of intestinal inflammation (5). Neither small nor large intestinal epithelial apoptotic cell numbers differed in conventionally colonized, ABx, and reconstituted mice at days 7 and 28 post-FMT (Figure 2A). Interestingly, quantification of Ki67 expression cells as a sensitive measure for cell proliferation and regeneration (34) revealed reduced numbers of proliferating cells in both ileal and colonic epithelia of ABx mice (*p* < 0.001; Figure 2B). As early as 7 days post-FMT, however, Ki67^+^ cell numbers reached basal counts again (*p* < 0.001 vs ABx; Figure 2B). Hence, neither broad-spectrum antibiotic treatment nor FMT results in increased intestinal apoptosis, whereas reconstitution with complex intestinal microbiota is essential for restoring cell proliferative and regenerative measures during physiological tissue turnover within the intestinal tract.

### Impact of Broad-Spectrum Antibiotic Therapy and Subsequent FMT on Innate and Adaptive Immune Cell Subsets in Murine Small and Large Intestines *In Situ*

**Figure 3:**
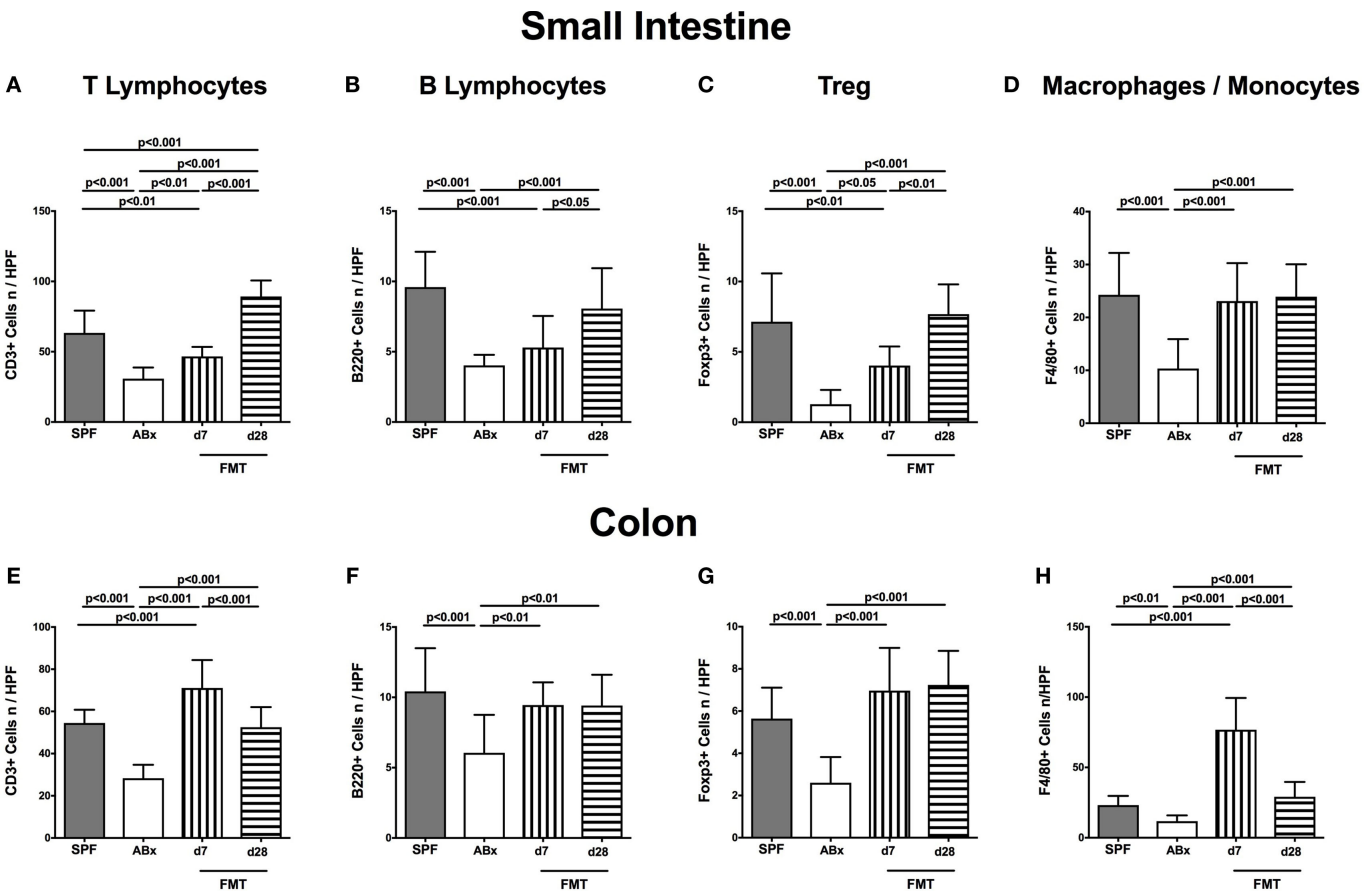
**Ileal and colonic immune cell populations in secondary abiotic and microbiota-reconstituted mice**. The average numbers of T lymphocytes [positive for CD3 **(A,E)**], B lymphocytes [positive for B220 **(B,F)**], regulatory T cells [Treg, positive for Foxp3 **(C,G)**], and macrophages/monocytes [positive for F4/80 **(D,H)**] from at least six representative high power fields (HPFs, 400× magnification) per animal were determined in *ex vivo* biopsies taken from the small intestine [upper panel **(A–D)**] and colon [lower panel **(E–H)**] of conventional mice (SPF, gray bars), secondary abiotic mice (ABx, white bars), and with microbiota-reconstituted abiotic mice at day (d) 7 (bars with vertical lines) or d28 (bars with horizontal lines) following fecal microbiota transplantation (FMT).

To examine the impact of the intestinal microbiota on abundances of distinct immune cell populations in the small and large intestines, we microscopically quantitated respective immune cell subsets in small intestinal and colonic paraffin sections applying *in situ* immunohistochemistry. In microbiota-depleted mice, significantly reduced numbers of CD3^+^ T lymphocytes (*p* < 0.001; Figures 3A,E), B220^+^ B lymphocytes (*p* < 0.001; Figures 3B,F), Foxp3^+^ regulatory T cells (Treg, *p* < 0.001; Figures 3C,G) as well as of F4/80^+^ monocytes and macrophages (*p* < 0.01–0.001; Figures 3D,H) in both ileum and colon as compared to conventional mice could be observed. Following FMT colonic, but not ileal numbers of respective immune cell populations increased back to counts observed in control mice as early as 7 days post-FMT (*p* < 0.01–0.001 vs ABx, Figures 3E,H). In small intestines, however, T and B lymphocytes as well as Treg numbers were lower at day 7 post-FMT as compared to SPF mice but reached comparable or even higher counts thereafter (Figures 3A,C). Hence, our data underline the essential association of the complex commensal microbiota and the repertoire of innate and adaptive immune cell populations in both the small and large intestines.

### Impact of Broad-Spectrum Antibiotic Treatment and Subsequent FMT on Distinct Lymphocyte Populations in Murine Intestinal and Systemic Compartments

To further elaborate the role of the intestinal microbiota on adaptive immunity in mucosal, peripheral, and systemic compartments, we isolated lymphocytes of the small and large intestinal LP, MLN, and spleen and analyzed defined immune cell populations by flow-cytometric analysis. Gating strategies are depicted in Figures S2A–F in Supplementary Material.

**Figure 4:**
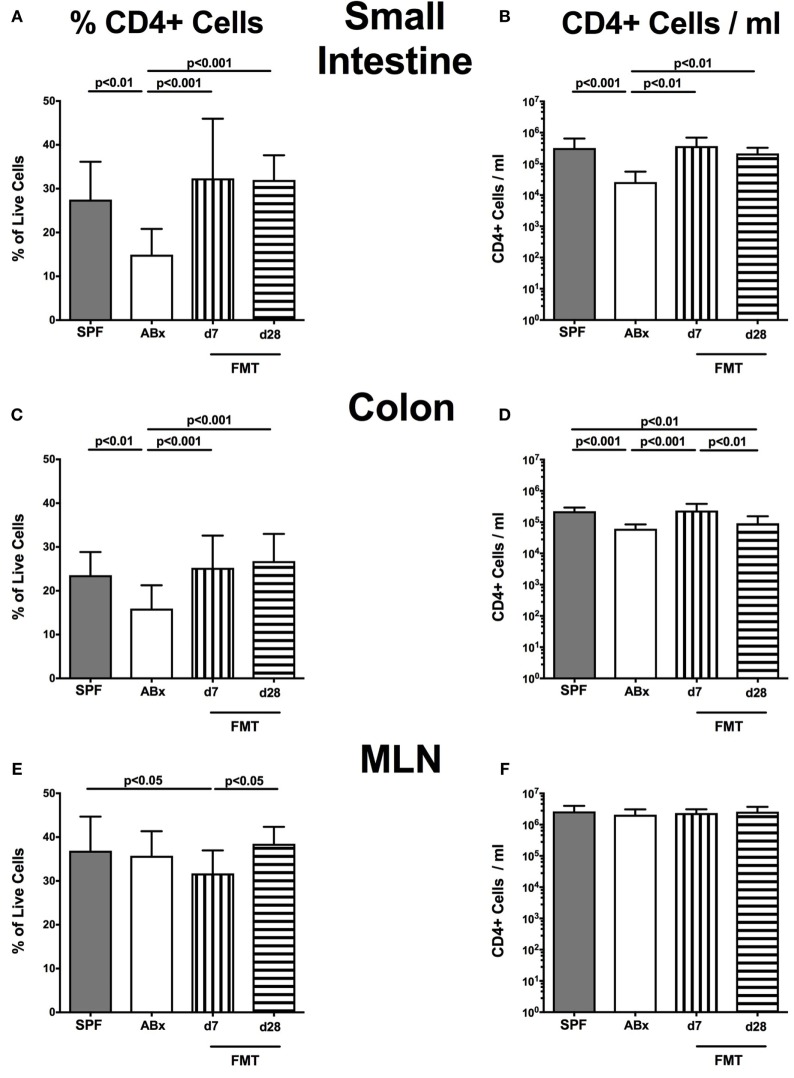

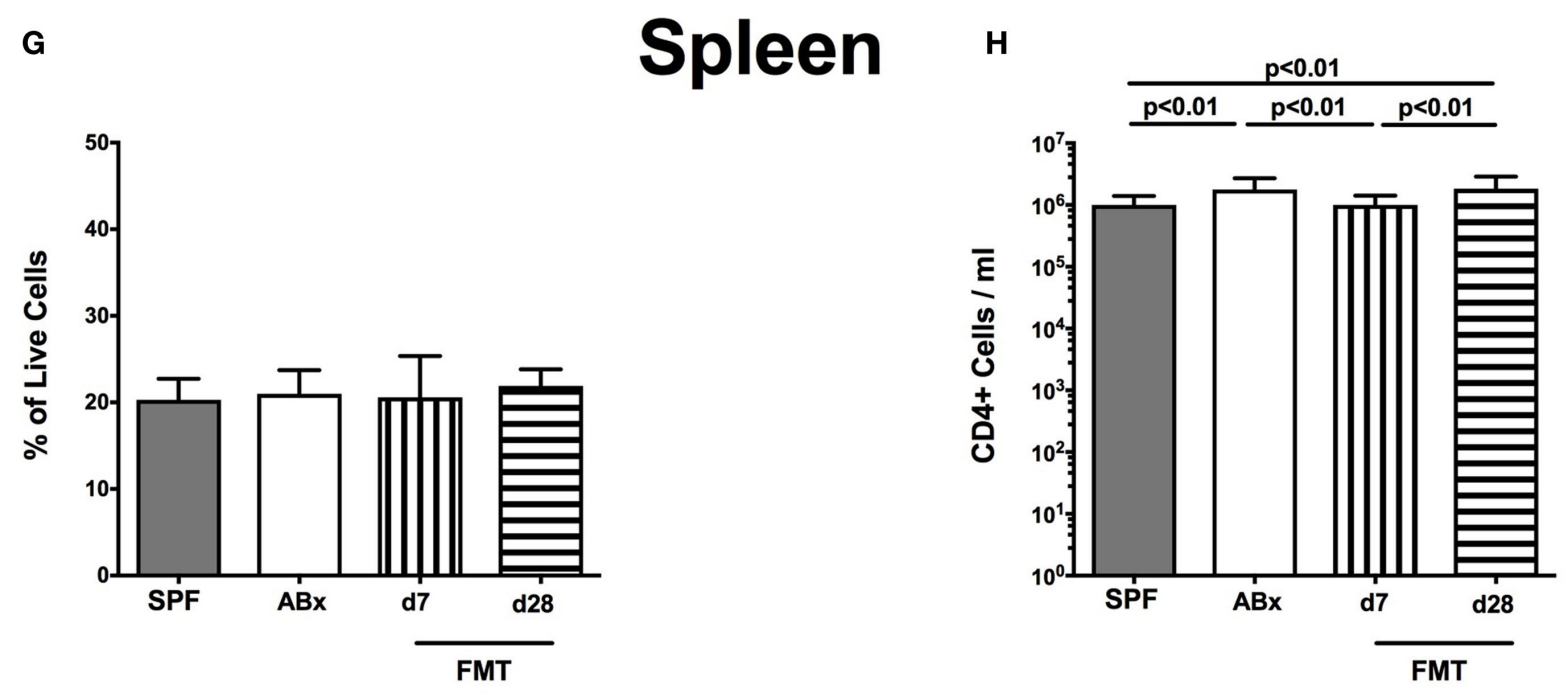
**CD4^+^ cells in intestinal and systemic compartments of secondary abiotic and microbiota-reconstituted mice**. The percentages [left panel **(A,C,E,G)**] and cell numbers [right panel **(B,D,F,H)**] of the CD4^+^ lymphocyte population within the small intestine **(A,B)**, colon **(C,D)**, mesenteric lymph nodes (MLN) **(E,F)**, and spleen **(G,H)** of naive conventional mice (SPF, gray bars), secondary abiotic mice (ABx, white bars), and recolonized mice on day (d) 7 (bars with vertical lines) and d28 (bars with horizontal lines) post-fecal microbiota transplantation (FMT) are depicted.

Broad-spectrum antibiotic treatment induced a significant reduction of both relative abundances and absolute numbers of CD4^+^ helper T lymphocytes in the small and large intestinal LP that could be restored at day 7 post-FMT, whereas colonic CD4^+^ cell concentrations further declined thereafter (*p* < 0.01–0.001; Figures 4A–D). Interestingly, within the MLN, but not other compartments, decreased percentages of CD4^+^ cells could be observed at day 7 post-FMT (*p* < 0.05; Figures 4E,F). In the spleen, frequencies of CD4^+^ cells were not affected by microbiota depletion (n.s.; Figure 4G). Notably, increased splenic CD4^+^ cell numbers were determined in ABx mice that slightly declined until day 7 post-FMT but increased to even supra-basal levels thereafter (*p* < 0.01; Figure 4H). These results point toward a potential “systemic accumulation” of these cells in the splenic compartment in absence of the intestinal microbiota.

**Figure 5:**
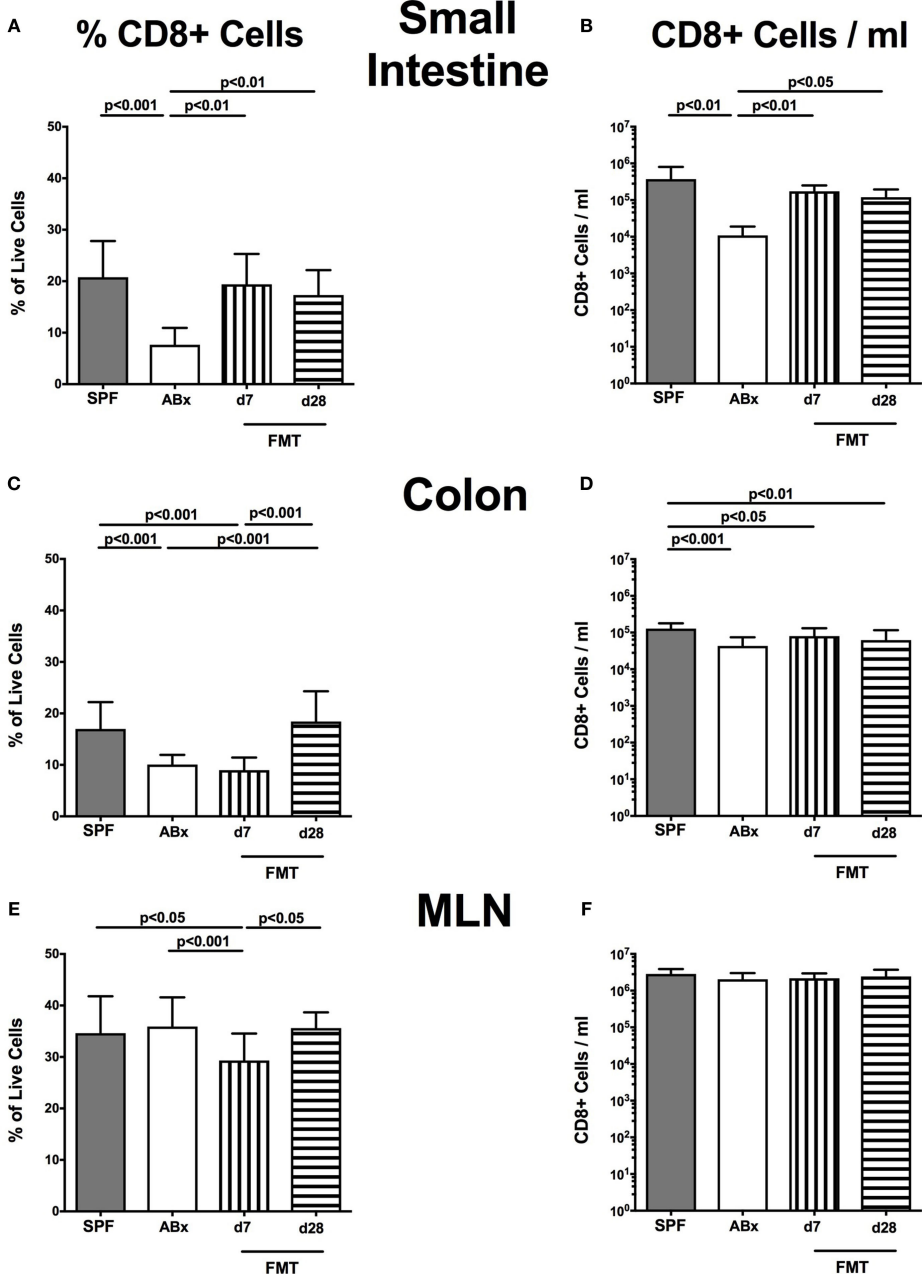

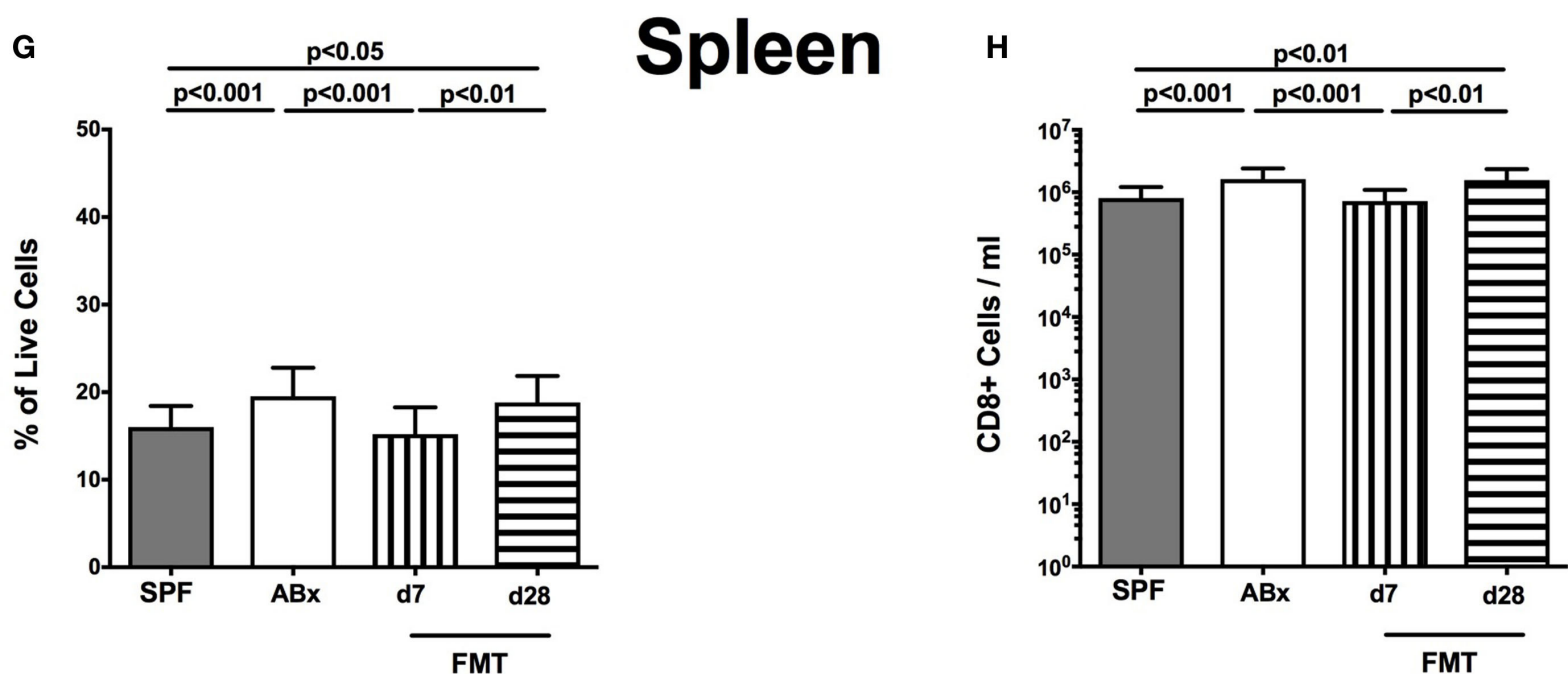
**CD8^+^ cells in intestinal and systemic compartments of secondary abiotic and microbiota-reconstituted mice**. The percentages [left panel **(A,C,E,G)**] and cell numbers [right panel **(B,D,F,H)**] of the CD8^+^ lymphocyte population within the small intestine **(A,B)**, colon **(C,D)**, mesenteric lymph nodes (MLN) **(E,F)**, and spleen **(G,H)** of naive conventional mice (SPF, gray bars), secondary abiotic mice (ABx, white bars), and recolonized mice at day (d) 7 (boxes with vertical lines) and d28 (bars with horizontal lines) post-fecal microbiota transplantation (FMT) are depicted.

In the small intestinal, LP decreased frequencies and cell numbers of CD8^+^ cytotoxic T cells were observed following microbiota depletion (*p* < 0.01–0.001; Figures 5A,B) that could be completely restored upon FMT. In the colonic LP, CD8^+^ cell frequencies were reduced upon antibiotic treatment and reestablished rather late following microbiota reconstitution (i.e., until day 28 post-FMT) (*p* < 0.001, d28 vs ABx; Figure 5C). Notably, colonic CD8^+^ cell numbers were profoundly affected by antibiotic treatment of mice, irrespective whether recolonized or not as indicated by lower counts as compared to SPF controls (*p* < 0.05–0.001; Figure 5D). In line with CD4^+^ cell numbers, CD8^+^ cells within MLN were not affected by antibiotic treatment and/or subsequent bacterial recolonization (n.s.; Figures 5E,F).

Relative abundances as well as absolute numbers of splenic CD8^+^ cells increased following antibiotic treatment (*p* < 0.05–0.001; Figures 5G,H), decreased as early as 7 days post-FMT but increased again 28 days after FMT to higher levels than in SPF mice.

We next addressed whether also B lymphocytes were affected following antibiotic treatment and restoration of the intestinal microbiota. In fact, also absolute numbers of B220^+^ cells decreased in small intestines, colon, and MLN upon microbiota depletion, but conversely increased in the spleen. FMT could sufficiently restore small intestinal, but not colonic B220^+^ cell counts as early as 7 days thereafter (Figures S3B,D in Supplementary Material). Again, microbiota depletion-induced elevation of splenic B220^+^ cells was reversed until day 7 post-FMT (*p* < 0.05; Figure S3H in Supplementary Material). Interestingly, proportions of B220^+^ B lymphocytes within small intestine, colon, and spleen were rather unaffected by the colonization status of mice (Figures S3A,C,G in Supplementary Material), whereas an increased proportion of B cells could be observed in MLN at day 7 post-FMT as compared to the other groups (*p* < 0.001; Figure S3E in Supplementary Material).

Taken together, these data point out that a virtual eradication of the intestinal microbiota by broad-spectrum antibiotic treatment affects both absolute numbers and relative abundances of distinct adaptive immune cell population not only locally (i.e., in the intestinal tract) but also has far-reaching consequences on systemic immune functions.

### Impact of Broad-Spectrum Antibiotic Treatment and Subsequent FMT on Memory/Effector T Cells, Treg, and Activated DC in Murine Intestinal and Systemic Compartments

**Figure 6:**
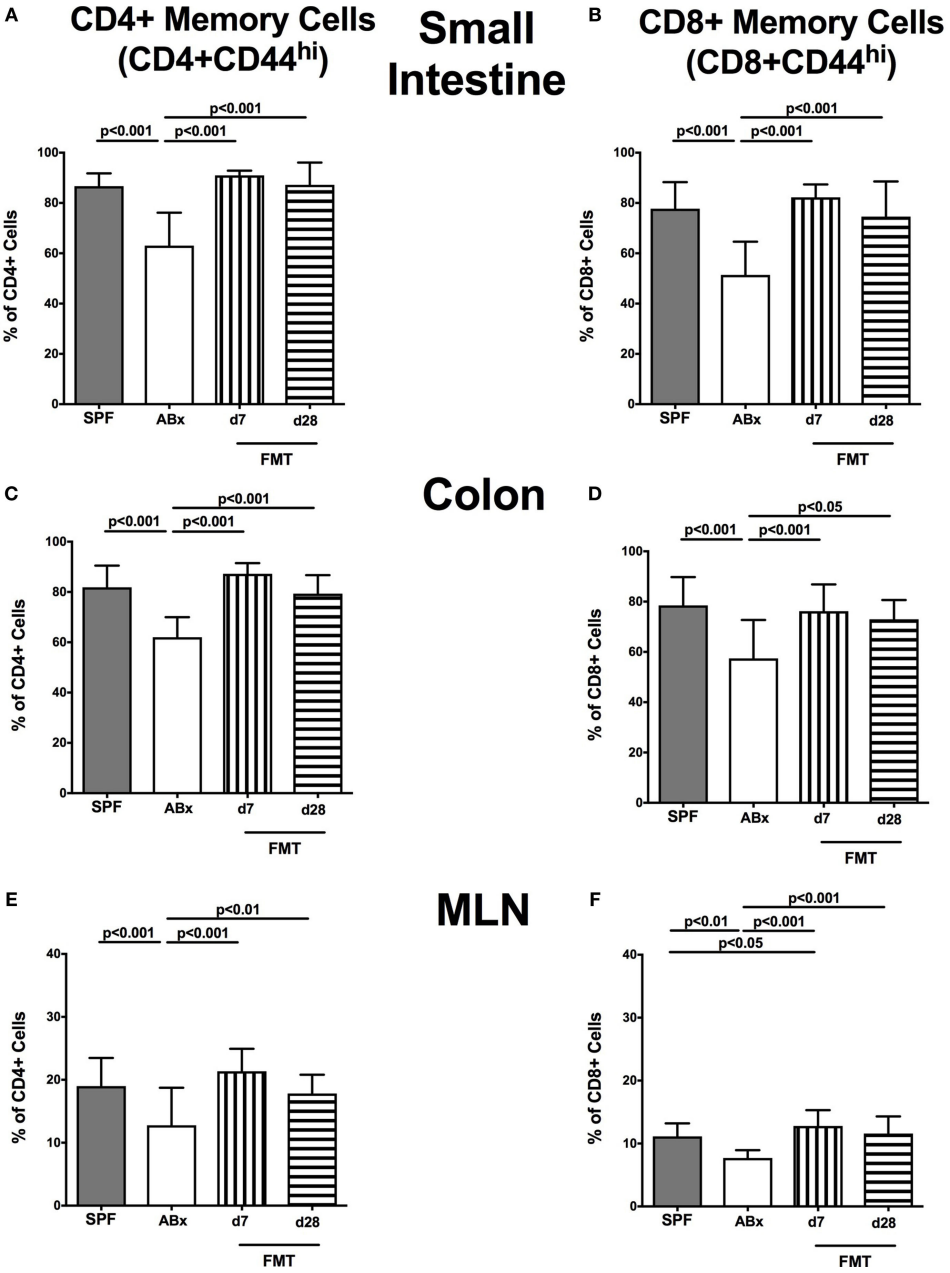

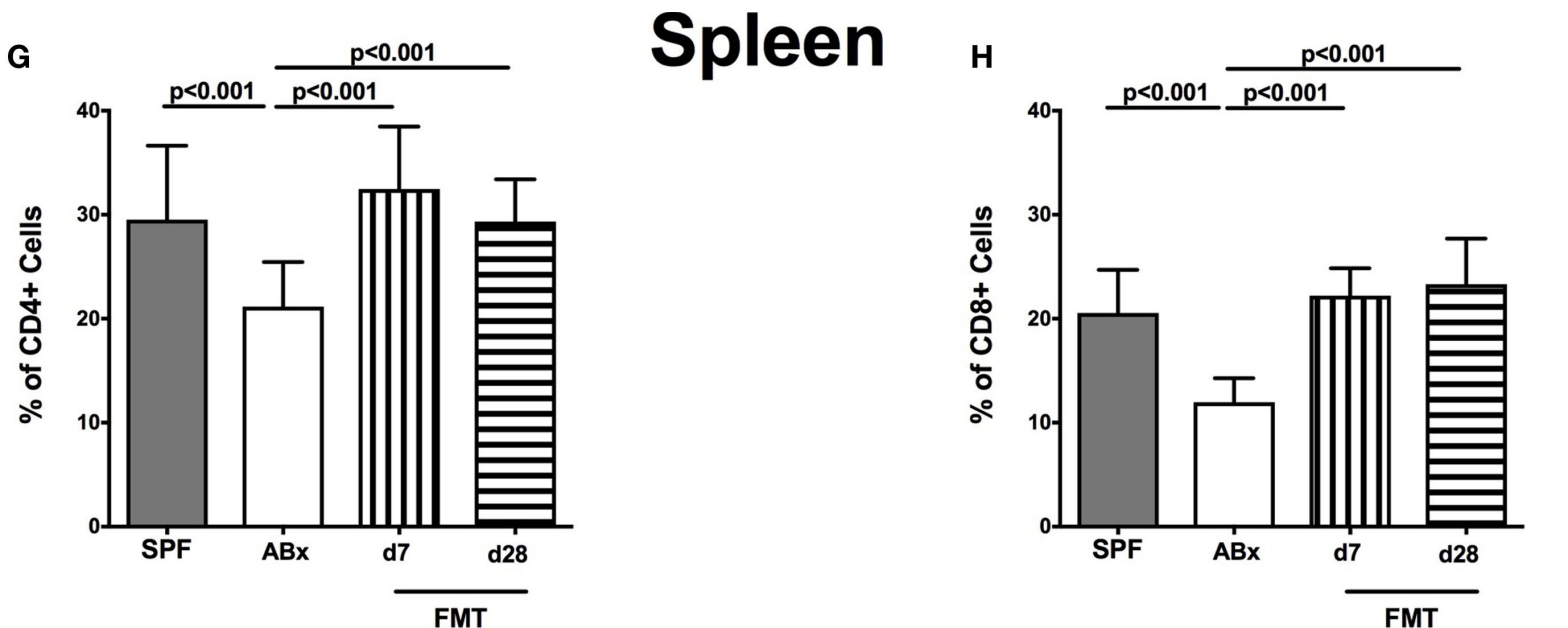
**Memory/effector T cell compartment in intestinal and systemic compartments of secondary abiotic and microbiota-reconstituted mice**. The proportions of CD4^+^ memory/effector cells [CD4^+^CD44^hi^, gated on CD4^+^ cells, left panel **(A,C,E,G)**] and CD8^+^ memory/effector cells [CD8^+^CD44^hi^, gated on CD8^+^ cells, right panel **(B,D,F,H)**] within the small intestine **(A,B)**, colon **(C,D)**, mesenteric lymph nodes (MLN) **(E,F)**, and spleen **(G,H)** of naive conventional mice (SPF, gray bars), secondary abiotic mice (ABx, white bars), and recolonized mice at day (d) 7 (boxes with vertical lines) and d28 (bars with horizontal lines) post-fecal microbiota transplantation (FMT) are depicted.

We next surveyed the impact of broad-spectrum antibiotic therapy and FMT on defined T cell subsets and on the activation status of distinct cell populations. To address this, the surface marker CD44 that is expressed upon antigen contact (35) on CD4^+^ and CD8^+^ cells was analyzed. Upon broad-spectrum antibiotic treatment, both CD44 positive CD4^+^ and CD8^+^ memory/effector cells were less abundant in all surveyed lymphoid compartments (*p* < 0.01–0.001; Figure 6). This effect could, however, be reversed upon FMT as indicated by higher abundances of CD44 positive CD4^+^ and CD8^+^ cells at days 7 and 28 post-FMT as compared to ABx mice (*p* < 0.05–0.001; Figure 6). At day 7 post-FMT, CD8^+^CD44^+^ in the MLN were even more abundant than in SPF mice but reached basal levels thereafter (*p* < 0.05; Figure 6F).

As already observed for the other immune cell subsets, also CD4^+^CD25^+^ Treg were strongly diminished in small and large intestines, MLN, and spleen of ABx mice (*p* < 0.01–0.001; Figures S4A,C,E,G in Supplementary Material), but this reduction could be reversed as early as 7 days following FMT (*p* < 0.001; Figures S4A,C,E,G in Supplementary Material). Interestingly, in all analyzed immunological compartments, Treg numbers were even higher at day 7 post-FMT as compared to naive mice but declined back to basal levels thereafter (*p* < 0.01–0.001; Figures S4A,C,E,G in Supplementary Material).

As for Treg, a strong downregulation of CD86 expression, a costimulatory molecule marking activated DC (36), could be observed in the small intestine, colon, MLN, and spleen upon broad-spectrum antibiotic treatment (*p* < 0.001; Figures S4B,D,F,H in Supplementary Material). Within 7 days following FMT, however, CD86^+^ cells reached basal levels again (Figures S4B,D,F,H in Supplementary Material). As already described for Treg, activated DC were even more abundant in spleens at day 7 post-FMT as compared to naive mice (*p* < 0.001; Figures S4H in Supplementary Material) but declined to basal levels thereafter.

Hence, memory/effector T cells, Treg, and activated DC are highly microbiota dependent, are disturbed following broad-spectrum antibiotic treatment, but can be restored upon FMT.

### Impact of Broad-Spectrum Antibiotic Treatment and Subsequent FMT on Cytokine Production in Murine Intestinal and Systemic Compartments

**Figure 7:**
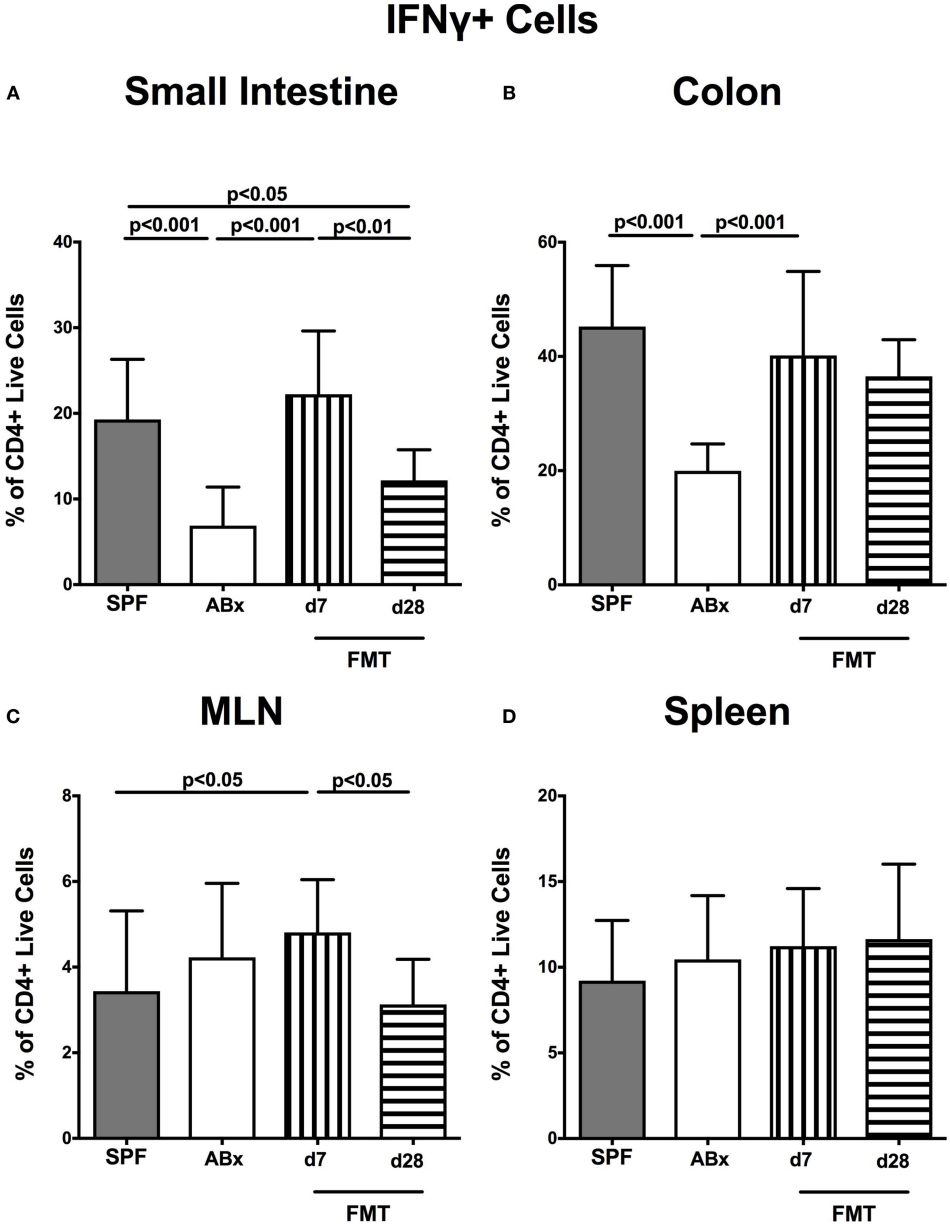
**IFN-γ-producing CD4^+^ cells in intestinal and systemic compartments of secondary abiotic and microbiota-reconstituted mice**. The percentages of IFN-γ-producing CD4^+^ cells in the **(A)** small intestine, **(B)** colon, **(C)** mesenteric lymph nodes (MLN), and **(D)** spleen of naive conventional mice (SPF, gray bars), secondary abiotic mice (ABx, white bars), and recolonized mice at day (d) 7 (boxes with vertical lines) and d28 (bars with horizontal lines) post-fecal microbiota transplantation (FMT) are depicted.

**Figure 8:**
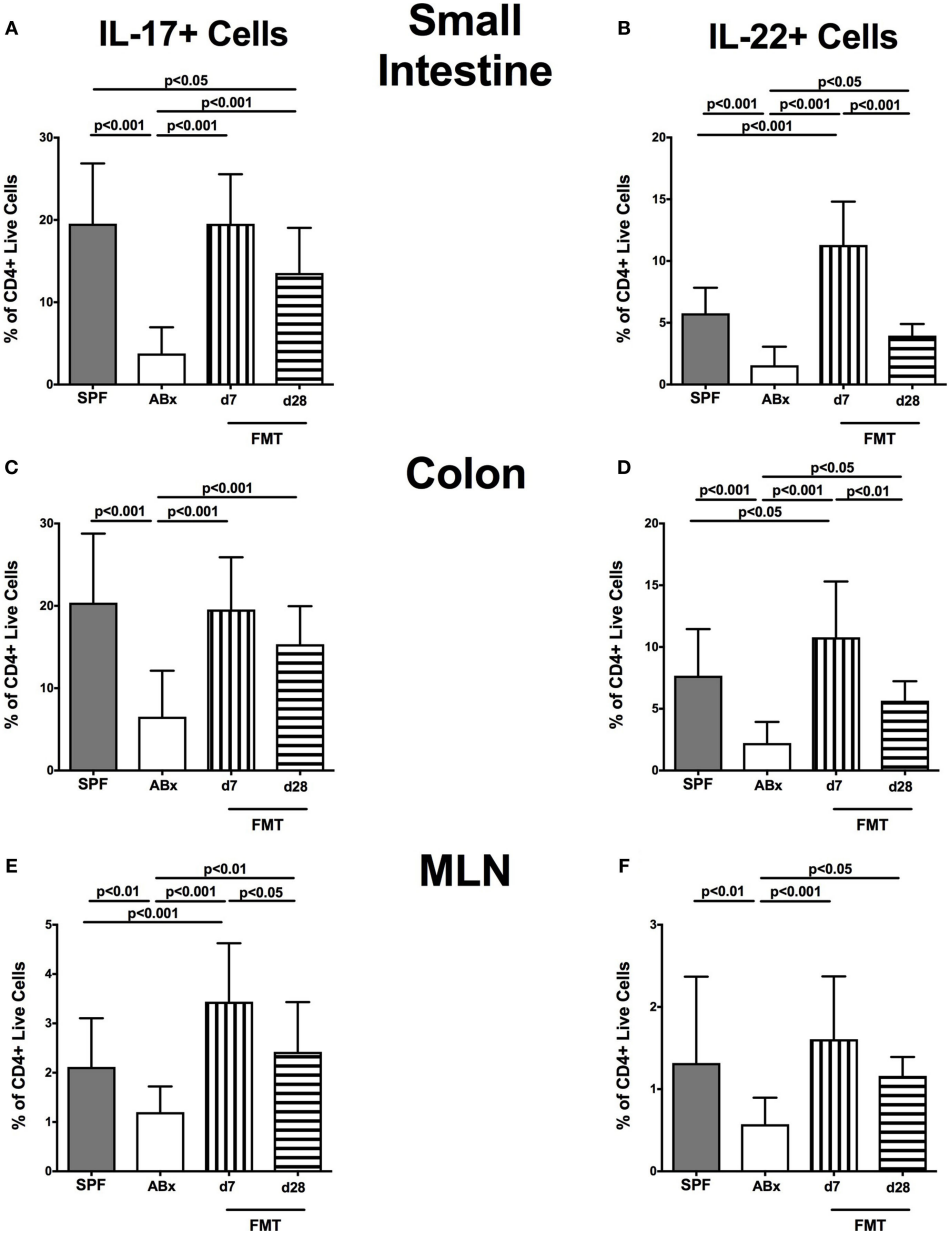

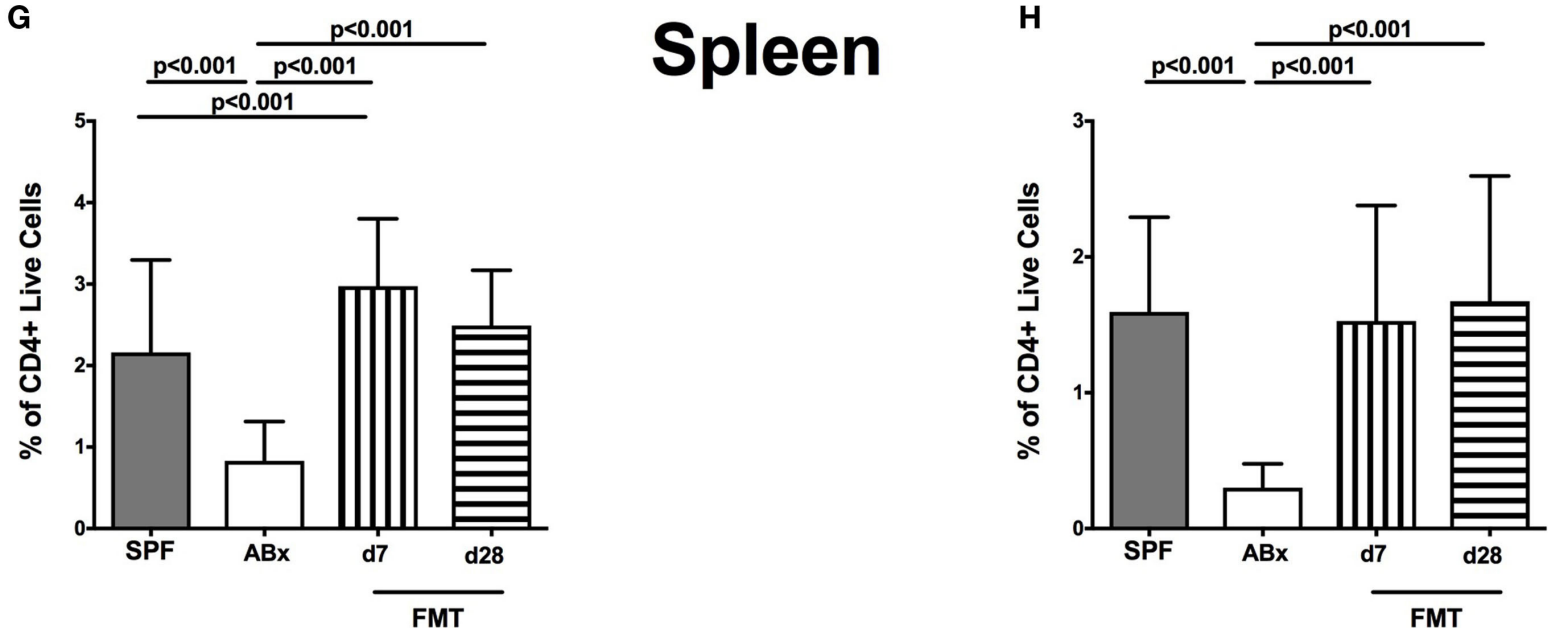
**IL-17- and IL-22-producing CD4^+^ cells in intestinal and systemic compartments of secondary abiotic and microbiota-reconstituted mice**. The percentages of IL-17- [left panel **(A,C,E,G)**] and IL-22- [right panel **(B,D,F,H)**] producing CD4^+^ cells in the small intestine **(A,B)**, colon **(C,D)**, mesenteric lymph nodes (MLN) **(E,F)**, and spleen **(G,H)** of naive conventional mice (SPF, gray bars), secondary abiotic mice (ABx, white bars), and recolonized mice at day (d) 7 (boxes with vertical lines) and d28 (bars with horizontal lines) post-fecal microbiota transplantation (FMT) are depicted.

**Figure 9:**
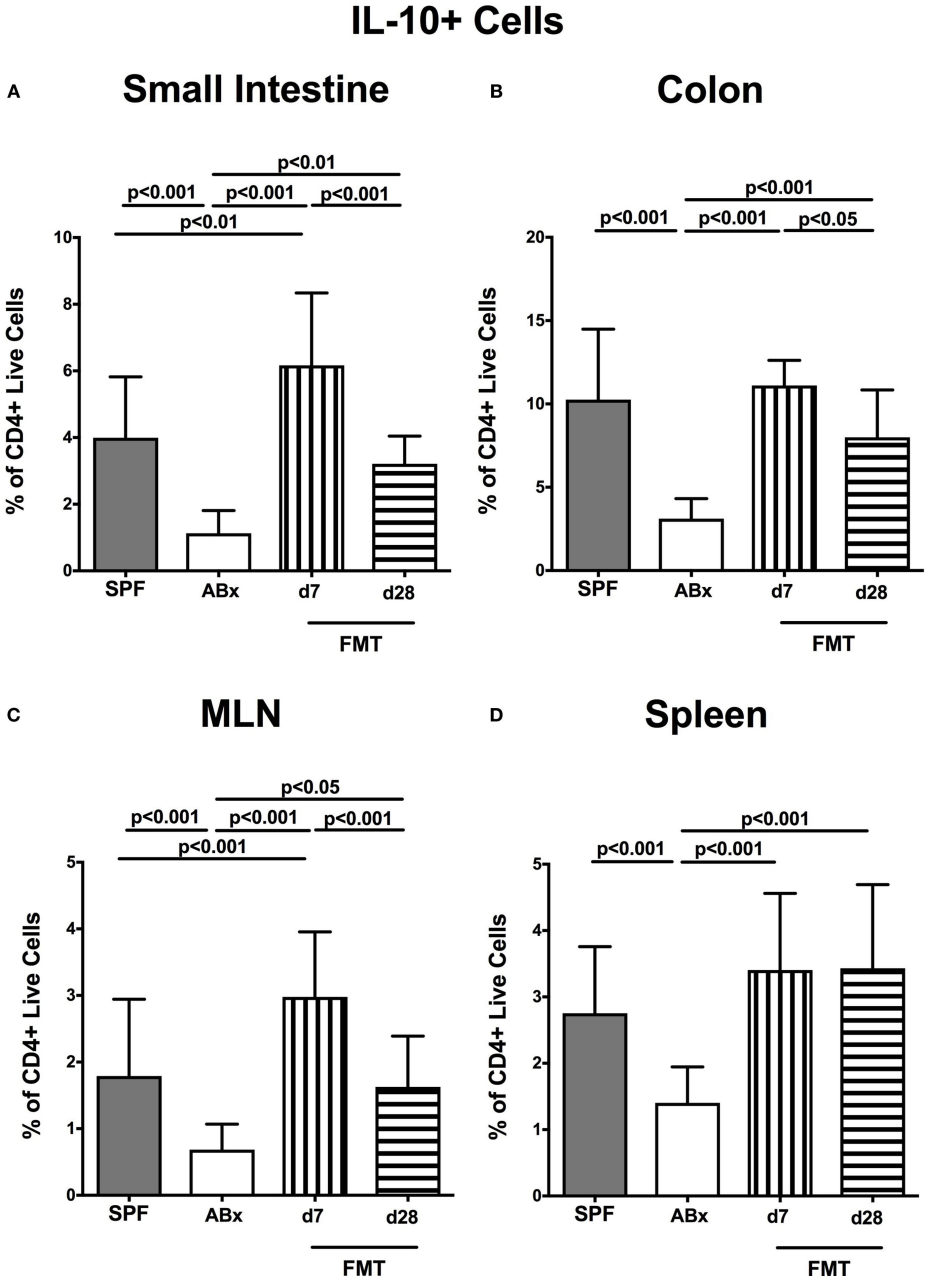
**IL-10-producing CD4^+^ cells in intestinal and systemic compartments of secondary abiotic and microbiota-reconstituted mice**. The percentages of IL-10-producing CD4^+^ cells in the **(A)** small intestine, **(B)** colon, **(C)** mesenteric lymph nodes (MLN), and **(D)** spleen of naive conventional mice (SPF, gray bars), secondary abiotic mice (ABx, white bars), and recolonized mice at day (d) 7 (boxes with vertical lines) and d28 (bars with horizontal lines) post-fecal microbiota transplantation (FMT) are depicted.

We further addressed whether microbiota depletion and subsequent reconstitution by FMT affected pro- and anti-inflammatory cytokine expression of CD4^+^ cells in intestinal and systemic lymphoid compartments by performing intracellular cytokine stainings. Gating strategies are depicted in Figures S2G–I in Supplementary Material. Decreased IFN-γ-expressing CD4^+^ cells were detected in small and large intestines of ABx mice but increased back to naive levels until day 7 following microbiota reconstitution (*p* < 0.001; Figures 7A,B). Of note, at day 7, but not day 28 following FMT, IFN-γ-expressing CD4^+^ cells were more prominent in MLN as compared to naive controls (*p* < 0.05; Figure 7C). Splenic CD4^+^IFN-γ^+^ cells, however, were virtually unaffected by antibiotic treatment and bacterial reconstitution (n.s.; Figure 7D). Notably, CD4^+^ cells expressing IL-17 and IL-22 were downregulated in small and large intestines, in MLN as well as in the spleen upon broad-spectrum antibiotic treatment but could be reversed as early as 7 days following FMT (*p* < 0.01–0.001; Figure 8). In addition, CD4^+^IL17^+^ cells in the MLN and spleen as well as small and large intestinal CD4^+^ IL-22^+^ cells were even higher at day 7 following FMT as compared to naive animals (*p* < 0.05–0.001; Figures 8B,D,E,G) but declined back to basal levels thereafter (*p* < 0.01–0.001). Furthermore, a strong reduction of CD4^+^ lymphocytes producing the anti-inflammatory cytokine IL-10 could be determined in all immunological sites following antibiotic therapy (*p* < 0.001; Figure 9). FMT could completely restore this cell population in all examined lymphoid compartments. Importantly, higher levels of IL-10 productions were observed in the small intestinal LP and MLN of mice at day 7 post-FMT than in their naive counterparts (*p* < 0.01–0.001; Figures 9A,C).

These findings were further supported by results obtained from mRNA analysis of respective cytokines measured in ileal and colonic *ex vivo* biopsies (Figure S5 in Supplementary Material). IFN-γ expression was downregulated upon antibiotic treatment in the small intestine only, an effect that was fully reversed until day 28 following FMT (Figure S5A in Supplementary Material). Moreover, antibiotic treatment resulted in strong suppression of both IL-17 and IL-22 mRNA expression. In the colon, expression of respective cytokines fully recovered as early as 7 days post-microbiota reconstitution, whereas small intestinal IL-17 mRNA levels measured at day 28 following FMT were approximately 300 times higher than in ABx mice, but still significantly lower as compared to naive SPF control mice (Figures S5B,C in Supplementary Material). In recolonized mice, a trend toward higher small intestinal expression of IL-22 mRNA could be observed (n.s.; Figures S5B,C in Supplementary Material). Furthermore ABx mice also displayed lower levels of IL-10 mRNA expression in both small and large intestines than their conventional counterparts (Figure S5D in Supplementary Material). At day 7 post-FMT, a strong IL-10 response could be only observed in the colon, whereas 28 days after microbiota reassociation, IL-10 expression reached naive levels in both compartments (Figure S5D in Supplementary Material). Hence, long-term broad-spectrum antibiotic treatment leads to suppression of both pro- and anti-inflammatory cytokines. These effects can, however, almost fully be reversed by recolonization with complex murine intestinal microbiota.

## Discussion

The complex mutualistic microbiota–host relationships, particularly the microbial stimuli-induced changes of immune cell homeostasis have elicited increased interest in recent years. Although isolator-raised GF mice have greatly advanced our understanding of the microbiota impact on the immune system, they do not address the role of microbiota-induced changes in host immunity later in life, given that profound immunological changes such as hypoplastic PP, reduced numbers of IgA-producing plasma cells (37), and LP CD4^+^ T cells (38), smaller germinal centers in MLN and poorly formed B and T cell zones in the spleens and lymph nodes could be shown in these mice (6, 7). Furthermore, increased evidence regarding pathologies related to antibiotic therapy [reviewed in Ref. (39)] including antibiotic-associated diarrhea (40), allergic inflammation (41), and asthma (42) has underlined the impact of antibiotics induced perturbations of the intestinal microbiota on host physiology.

In the present study, we applied a mouse model suitable to dissect the interplay between microbiota, antibiotics, and host immune system in conventionally raised and developed mice. Following broad-spectrum antibiotic treatment secondary abiotic (i.e., gnotobiotic), mice were virtually lacking the intestinal microbiota as shown by cultural as well as by highly sensitive culture-independent molecular methods (5, 24, 43). Most strikingly, quantitative 16S rRNA based real-time PCR analysis presented here revealed that gene copy numbers of the main bacterial intestinal groups did not differ between fecal samples taken from secondary abiotic mice and autoclaved food pellets. Notably, it is rather impossible to differentiate whether detected 16S rRNA gene copies in ABx mice were derived from avital (“dead”) or viable (fastidious/uncultivable) bacterial cells. Hence, secondary abiotic mice provide the following advantages [as reviewed in Ref. (43)]: first, they circumvent the developmental anomalies of isolator-raided GF mice and offer second, the opportunity to analyze the antibiotics induced disruption of the commensal intestinal microbiota composition and subsequent adverse consequences (“collateral damages”) for host immunity. Third, upon cessation of antibiotic treatment, secondary abiotic mice can be stably reassociated with single bacterial species, with a combination of defined commensals or pathogens or with a complex commensal microbiota derived from mice or even a different host including humans (5, 24, 43). Finally, neither secondary abiotic nor with complex microbiota reassociated mice display any adverse clinical sequelae such as wasting or diarrhea or microscopic signs of intestinal inflammation including epithelial apoptosis. We could further confirm that the intestinal microbiota composition was stable for at least 4 weeks following FMT and similar to conventionally colonized naive mice.

The reintroduction of complex microbiota into the host *via* FMT is a well-known therapy dating back to the Chinese Dong-jin dynasty in the fourth century (44) and has undergone a renaissance recently as a therapeutic option for the treatment of recurrent and refractory *C. difficile* toxin induced acute necrotizing pseudo-membranous enterocolitis (45–49).

Initial small and large intestinal *in situ* analysis of distinct innate as well as adaptive immune cell populations revealed that macrophages and monocytes as well as T lymphocytes, Treg, and B lymphocytes, respectively, were drastically reduced following antibiotic therapy but could be restored by FMT in a time-dependent fashion. This reinforced the microbiota dependent dynamics of mucosal immune cell homeostasis as prior evidence has revealed for both innate (22, 50) and adaptive immune responses (51, 52).

These data were supported by more detailed flow-cytometric analyses of lymphocytes isolated from different intestinal compartments including the small intestine, colon, and MLN. Overall, broad-spectrum antibiotics decreased distinct immune cell subsets such as Th cells, cytotoxic T cells, memory and effector T cells, B lymphocytes, Treg as well as activated DC, whereas reintroduction of the complex microbiota could sufficiently reverse the immune-depressive effects exerted by the antibiotic compounds. We could further observe, however, that immunological sites of the different levels (i.e., local/intestinal, extra-intestinal/systemic) do not always respond in the same manner and to a comparable extent to the absence of or reassociation with intestinal microbiota. For instance, a decline of CD4^+^, CD8^+^, and B220^+^ cell numbers in the small and large intestinal LP following antibiotic therapy was conversely associated with an increase of the respective immune cell populations in the spleen, pointing toward a possible centralization of lymphocytes due to missing interactions with bacterial antigens in the intestinal tract. Moreover, our data suggest an inverse relationship of the mentioned lymphocytic cell subsets between colon and spleen at different time points post-FMT, given that a colonic decrease of CD4^+^ and B220^+^ cells was, conversely, paralleled by an increase of the respective cell types at day 28 post-FMT in the spleen. One therefore needs to take into consideration that, while tempting to develop novel approaches to conveniently manipulate gut microbiota, changes in immune cell populations are not restricted to local, i.e., intestinal sites but might also lead to global/systemic consequences. This is also supported by previous studies with probiotic strains such as *Lactobacillus reuteri* 100-23 inducing systemic anti-inflammatory IL-10 production (53) or *Lactobacillus casei* (DN-114 001) alleviating skin inflammation (54).

One explanation for kinetic differences in reconstituting cell types following FMT of ABx mice could be that a minimum of time is required to fully compensate for the prominent collateral damages to the intestinal ecosystem and immune system that were caused by long-term antibiotic treatment. To accomplish this following FMT, the bacteria need to allocate niches, redevelop an intraluminal equilibrium for both bacteria–bacteria and microbiota–immune cell interactions. Together with data showing long-lasting consequences of antibiotic therapy on the human gut ecosystem (55, 56), these findings emphasize the need for considering long-term effects on immunity in patients undergoing antibiotic treatment.

Strikingly, recolonization with complex intestinal microbiota could not sufficiently recover CD8^+^ cell numbers in the colonic LP, suggesting that antibiotic treatment affects this cell population through commensal-independent mechanisms. While underlying mechanisms still need to be unraveled, this would fit with prior data already describing microbiota-independent immunomodulatory effects of antibiotic compounds such as macrolides (57) and fluoroquinolones (58). Immunomodulatory properties of macrolides were especially recognized due to their effectiveness in treating diffuse panbronchitis, a complex pulmonary disorder afflicting mainly East Asians (59), and have been confirmed by numerous *in vitro* and *in vivo* experiments. In mammalian host cells, for instance, macrolides impact the mitogen-activated protein kinase, extracellular signal-regulated kinase 1/2 (ERK 1/2) and nuclear factor-kappa B (NF-κB) pathways subsequently leading to inhibition of mucus secretion, suppression of the production and secretion of pro-inflammatory cytokines, inhibition of cell proliferation, suppression of iNOS-mediated NO production, and inhibition of chemotaxis [as reviewed in Ref. (57, 59)]. Moreover, *in vitro* experiments revealed that the fluoroquinolone moxifloxacin decreased the TNF and IL-1 production by lipopolysaccharide (LPS)-stimulated human monocytes (60). Fluoroquinolones have also been shown to protect mice from both lethal and sublethal LPS challenges by significantly reducing serum levels of pro-inflammatory cytokines such as IL-6, IL-12, and TNF (61, 62). Evidence suggests that fluoroquinolones affect the intracellular cyclic adenosine-3,5-monophosphate and phosphodiesterases as well as transcription factors such as NF-κB, activator protein 1, and NF of activated T cells [as reviewed in Ref. (58)].

Remarkably, one study revealed that two-thirds of intestinal gene expression alterations in antibiotic-treated mice occur microbiota independently, particularly affecting mitochondrial genes coding for electron transport chains, oxidation-reduction, ATP biosynthesis, and cellular and mitochondrial ribosomes (63).

In terms of activation status and cytokine profiling of immune cell populations, we could observe a rather different situation. Antibiotic treatment resulted in a strong reduction of Treg, activated DC, and of CD4^+^ and CD8^+^ memory/effector cells in all examined immunological sites, whereas a virtually complete recovery of these cell populations could be observed upon recolonization with complex microbiota, given that at day 28 post-FMT, the overall situation resembled that of naive untreated mice. Similarly, antibiotic-treated mice revealed declined production of IFN-γ in colon and ileum, and of IL-17, IL-22, and IL-10 in small and large intestines, MLN, and spleen. These effects could, however, be completely restored following FMT. Our data are well in line with previous findings stating the importance of microbiota-driven signaling for the expansion of cytokine-producing CD4^+^ cells in the gut (64). Moreover, it has been shown that fecal reassociation of GF mice simultaneously drives pro-inflammatory and regulatory immune responses (65). Taken together, these results emphasize the indispensable importance of the intestinal microbiota for differentiation of immune cells and maintenance of immune system homeostasis and confirm their capacity of restoring several impairments following antibiotic treatment. Furthermore, the antibiotics induced reduction of the Th17 cell compartment, which is important in protection against bacterial and fungal pathogens, particularly those encountered at mucosal surfaces (66), is well in line with the increased susceptibility of microbiota-depleted mice to pathogens (67, 68). This increased susceptibility to infection and inflammation may also be further amplified by the observed lower IL-10 levels in the lymphoid compartments with subsequent consequences for anti-inflammatory Treg-mediated responses.

### Summary and Conclusion

In the present study, we have focused on the effects of a complex murine microbiota on the immune system following antibiotics induced impairments not only of the intestinal ecosystem but also of peripheral as well as systemic immune functions. Whether the here displayed beneficial restoring effects exerted by reintroduced microbial antigens are due to the large bacterial loads, complexity and/or diversity of the introduced complex microbiota, or whether distinct species in concert with each other play a more important role in an orchestrated fashion, with the host immune system as the conductor, should be unraveled in more detail, but appears literally rather as a search for the needle in the hay stack. Nevertheless, it remains an outstanding and challenging issue to characterize the effects of single species and their products on the balance between pro-inflammatory and regulatory immune responses.

We are all aware of the fact that a rational and responsible antibiotic treatment is unavoidable under specific clinical conditions, but it is crucial to keep the effects of this therapy on the immune system in mind. These effects might be due to potential immune-modulating properties of the antimicrobial compound itself and/or due to microbiota-modulating (-depleting) sequelae of therapy or prophylaxis. Further knowledge of the orchestrated microbiota–host interplay could offer valuable contributions to the development of novel therapeutic approaches including strategies to enhance immunity and manipulating microbiota composition toward more beneficial (i.e., probiotic) species.

## Ethics Statement

All animal experiments were carried out according to the European Guideline for animal welfare (2010/63/EU) with approval of the commission for animal experiments headed by the “Landesamt für Gesundheit und Soziales” (LaGeSo, Berlin, Germany, registration numbers G0097/12 and G0184/12). Animal welfare was examined twice daily by assessment of clinical conditions.

## Author Contributions

IE performed experiments, analyzed data, and wrote paper. EK, UF, and UE performed experiments, analyzed data, and co-edited paper. CN and PB suggested critical parameters in design of experiments and supplied antibodies. AS provided advice in design and performance of experiments. AK analyzed data and co-edited paper. SB provided advice in design and performance of experiments and co-edited paper. MH designed and performed experiments, analyzed data, and co-wrote paper.

## Conflict of Interest Statement

The authors declare that the research was conducted in the absence of any commercial or financial relationships that could be construed as a potential conflict of interest.

## Supplementary Material

Figure S1 **Intestinal microbiota of conventional and reconstituted secondary abiotic mice**. At day (d) 7 (open squares; *n* = 16) and d28 (black squares; *n* = 18) following fecal microbiota transplantation, the intestinal microbiota composition was assessed in fecal samples applying quantitative real-time PCR amplifying variable regions of the bacterial 16S rRNA gene. The following main intestinal bacterial groups were determined (expressed as 16S rRNA gene numbers per nanogramg DNA): total eubacterial load (TL), enterobacteria (EB), enterococci (EC), lactic acid bacteria (LB), bifidobacteria (BIF), *Bacteroides/Prevotella* spp. (BP), *Clostridium coccoides* group (CLOCC), *Clostridium leptum* group (CLEP), and *Mouse Intestinal Bacteroides* (MIB). Conventionally colonized mice served as naive controls (N, black circles; *n* = 13).

Figure S2 **Gating strategies of extracellular stainings on one representative splenic sample after gating for lymphocytes and including only single cells**. **(A)** Exclusion of dead cells *via* LIVE/DEAD Fixable Aqua Dead Cell Stain kit (L/D) in the forward scatter, **(B)** CD4^+^ and CD8^+^ cells gated on living cells, **(C)** B cells and activated DC gated on CD4^−^CD8^−^ cells, **(D)** regulatory T cells, and **(E)** CD4^+^ memory/effector cells gated on CD4^+^ cells, **(F)** CD8^+^ memory/effector cells gated on CD8^+^ cells. Gating strategies of intracellular stainings on one representative ileal sample after gating for lymphocytes and including only single cells. **(G)** Identification of CD4^+^ living cells by exclusion of dead cells *via* LIVE/DEAD Fixable Aqua Dead Cell Stain kit (L/D), **(H)** IFN-γ and IL-10 gating, and **(I)** IL-17 and IL-22 gating.

Figure S3 **B220^+^ cells in intestinal and systemic compartments of secondary abiotic and microbiota-reconstituted mice**. The percentages [left panels **(A,C,E,G)**] and cell numbers [right panels **(B,D,F,H)**] of the B220^+^ lymphocyte population within the small intestine **(A,B)**, colon **(C,D)**, mesenteric lymph nodes (MLN) **(E,F)**, and spleen **(G,H)** of naive conventional mice (SPF, gray bars), secondary abiotic mice (ABx, white bars), and recolonized mice at day (d) 7 (boxes with vertical lines) and d28 (bars with horizontal lines) post-fecal microbiota transplantation (FMT) are depicted.

Figure S4 **Regulatory T cells (Treg) and activated DC in intestinal and systemic compartments of secondary abiotic and microbiota-reconstituted mice**. The frequencies of Treg (CD4^+^ CD25^+^, gated on CD4^+^ cells) [left panels **(A,C,E,G)**] and activated DC [CD86^+^, gated on CD4^−^CD8^−^ live cells, right panels **(B,D,F,H)**] in the small intestine **(A,B)**, colon **(C,D)**, mesenteric lymph nodes (MLN) **(E,F)**, and spleen **(G,H)** of naive conventional mice (SPF, gray bars), secondary abiotic mice (ABx, white bars), and recolonized mice at day (d) 7 (boxes with vertical lines) and d28 (bars with horizontal lines) post-fecal microbiota transplantation (FMT) are depicted.

Figure S5 **mRNA analysis of pro- and anti-inflammatory cytokines in small intestinal and colonic tissue**. RT-PCR results of **(A)** IFN-γ, **(B)** IL-17, **(C)** IL-22, and **(D)** IL-10 expression in small intestinal (upper panel) and colonic (lower panel) tissues derived from naive conventional mice (SPF, gray bars), secondary abiotic mice (ABx, white bars), and recolonized mice at day (d) 7 (boxes with vertical lines) and d28 (bars with horizontal lines) following fecal microbiota transplantation (FMT) are depicted.

## Abbreviations

ABx, secondary abiotic; BSA, bovine serum albumin; CFU, colony-forming units; DC, dendritic cells; DTE, dithioerythritol; EDTA, ethylenediaminetetraacetic acid; FCS, fetal calf serum; FMT, fecal microbiota transplantation; GF, germfree; HBSS, Hanks’ balanced salt solution; HPF, high power field; IBD, inflammatory bowel disease; LPL, lamina propria lymphocytes; LPS, lipopolysaccharide; MLN, mesenteric lymph nodes; PBS, phosphate-buffered saline; PFA, paraformaldehyde; PP, Peyer’s patches; PMA, phorbol myristate acetate; SPF, specific-pathogen-free; Th, T helper; TNBS, trinitrobenzenesulphonic acid; Treg, regulatory T cells.
