# Supplementary figures and images for: Immune Responses to Broad-Spectrum Antibiotic Treatment and Fecal Microbiota Transplantation in Mice
Source: Front Immunol. 2017 Apr 19;8:397. doi: 10.3389/fimmu.2017.00397 (PMC5395657; doi:10.3389/fimmu.2017.00397)

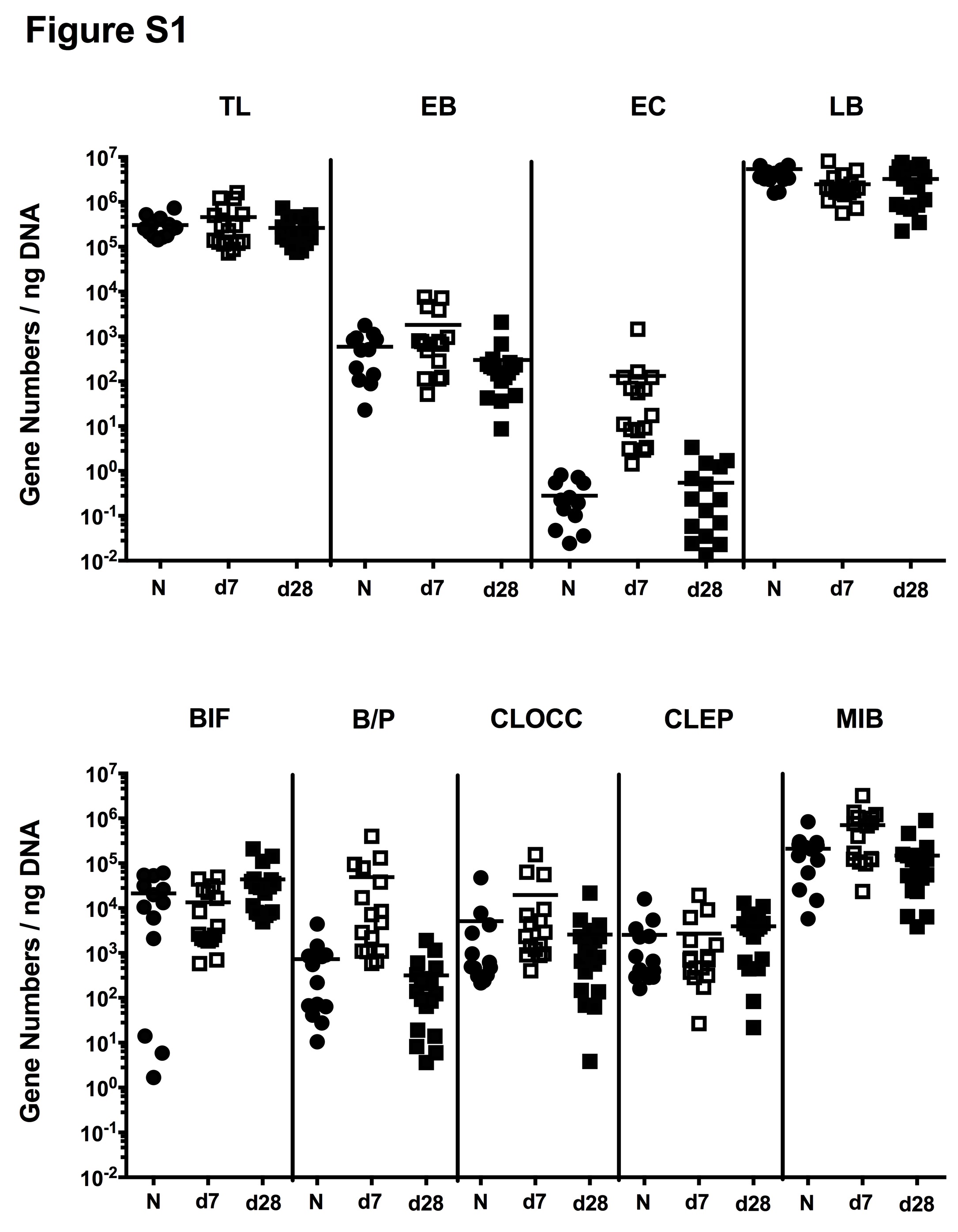

Supplement: Figure S1 — Intestinal microbiota of conventional and reconstituted secondary abiotic mice. At day (d) 7 (open squares; n = 16) and d28 (black squares; n = 18) following fecal microbiota transplantation, the intestinal microbiota composition was assessed in fecal samples applying quantitative real-time PCR amplifying variable regions of the bacterial 16S rRNA gene. The following main intestinal bacterial groups were determined (expressed as 16S rRNA gene numbers per nanogramg DNA): total eubacterial load (TL), enterobacteria (EB), enterococci (EC), lactic acid bacteria (LB), bifidobacteria (BIF), Bacteroides/Prevotella spp. (BP), Clostridium coccoides group (CLOCC), Clostridium leptum group (CLEP), and Mouse Intestinal Bacteroides (MIB). Conventionally colonized mice served as naive controls (N, black circles; n = 13). [file image_1.jpeg]

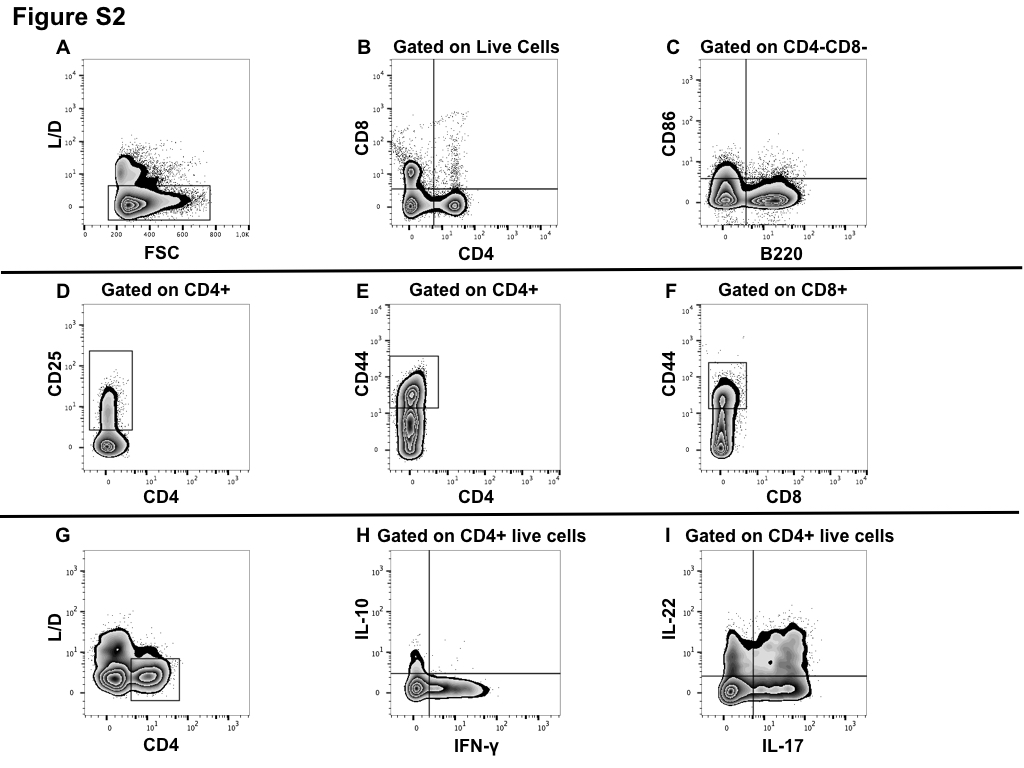

Supplement: Figure S2 — Gating strategies of extracellular stainings on one representative splenic sample after gating for lymphocytes and including only single cells. (A) Exclusion of dead cells via LIVE/DEAD Fixable Aqua Dead Cell Stain kit (L/D) in the forward scatter, (B) CD4+ and CD8+ cells gated on living cells, (C) B cells and activated DC gated on CD4−CD8− cells, (D) regulatory T cells, and (E) CD4+ memory/effector cells gated on CD4+ cells, (F) CD8+ memory/effector cells gated on CD8+ cells. Gating strategies of intracellular stainings on one representative ileal sample after gating for lymphocytes and including only single cells. (G) Identification of CD4+ living cells by exclusion of dead cells via LIVE/DEAD Fixable Aqua Dead Cell Stain kit (L/D), (H) IFN-γ and IL-10 gating, and (I) IL-17 and IL-22 gating. [file image_2.jpeg]

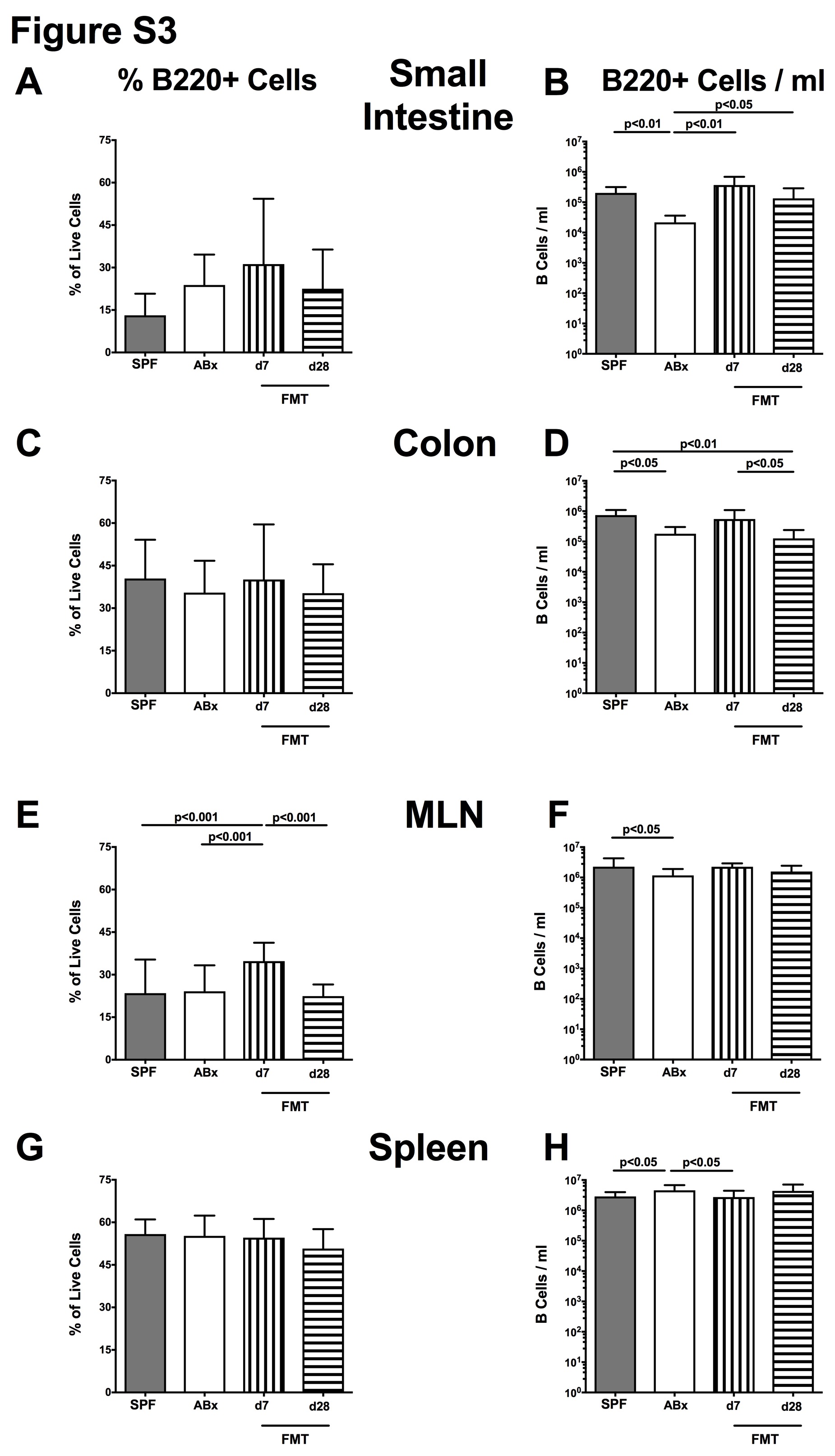

Supplement: Figure S3 — B220+ cells in intestinal and systemic compartments of secondary abiotic and microbiota-reconstituted mice. The percentages [left panels (A,C,E,G)] and cell numbers [right panels (B,D,F,H)] of the B220+ lymphocyte population within the small intestine (A,B), colon (C,D), mesenteric lymph nodes (MLN) (E,F), and spleen (G,H) of naive conventional mice (SPF, gray bars), secondary abiotic mice (ABx, white bars), and recolonized mice at day (d) 7 (boxes with vertical lines) and d28 (bars with horizontal lines) post-fecal microbiota transplantation (FMT) are depicted. [file image_3.jpeg]

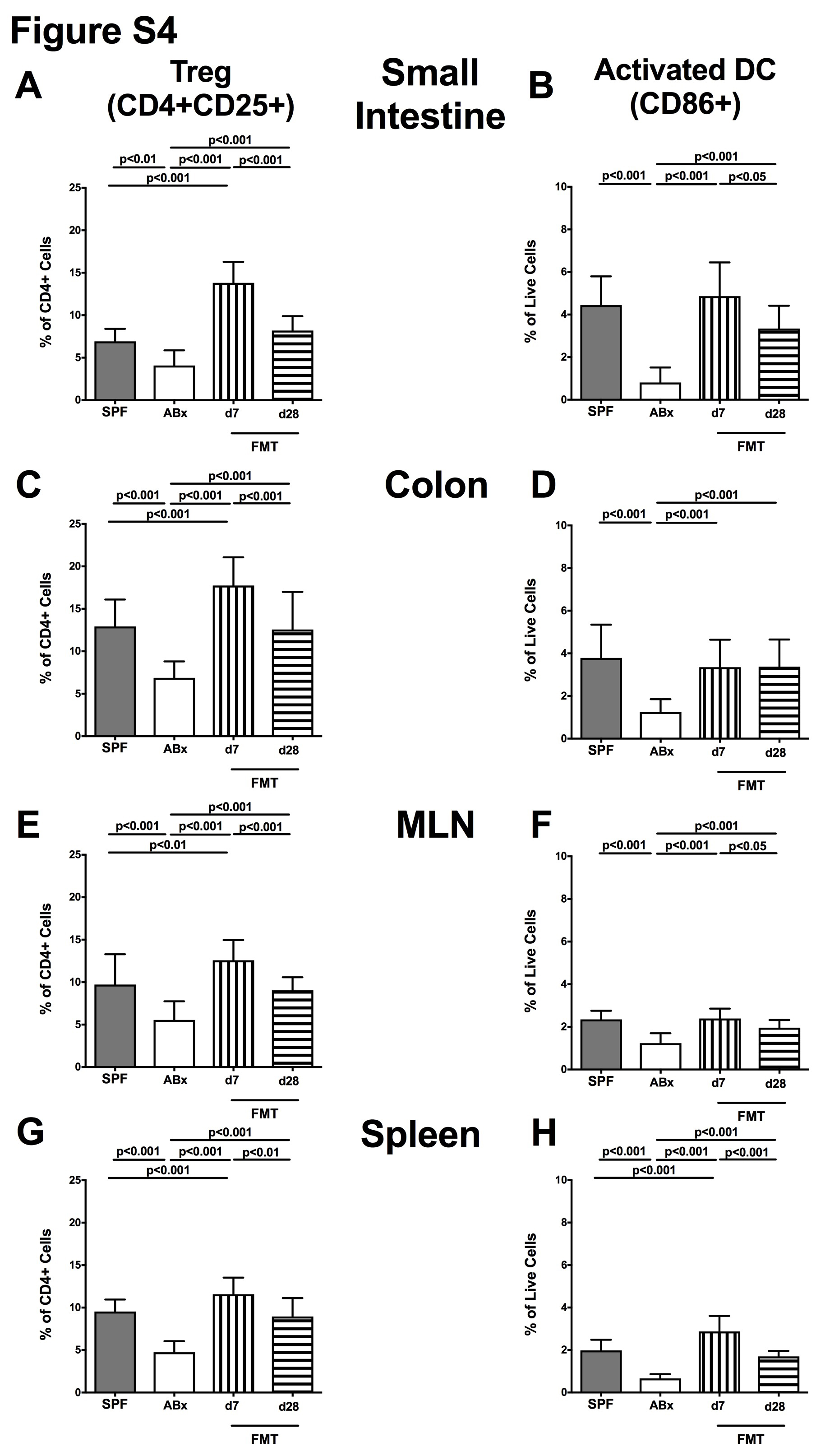

Supplement: Figure S4 — Regulatory T cells (Treg) and activated DC in intestinal and systemic compartments of secondary abiotic and microbiota-reconstituted mice. The frequencies of Treg (CD4+ CD25+, gated on CD4+ cells) [left panels (A,C,E,G)] and activated DC [CD86+, gated on CD4−CD8− live cells, right panels (B,D,F,H)] in the small intestine (A,B), colon (C,D), mesenteric lymph nodes (MLN) (E,F), and spleen (G,H) of naive conventional mice (SPF, gray bars), secondary abiotic mice (ABx, white bars), and recolonized mice at day (d) 7 (boxes with vertical lines) and d28 (bars with horizontal lines) post-fecal microbiota transplantation (FMT) are depicted. [file image_4.jpg]

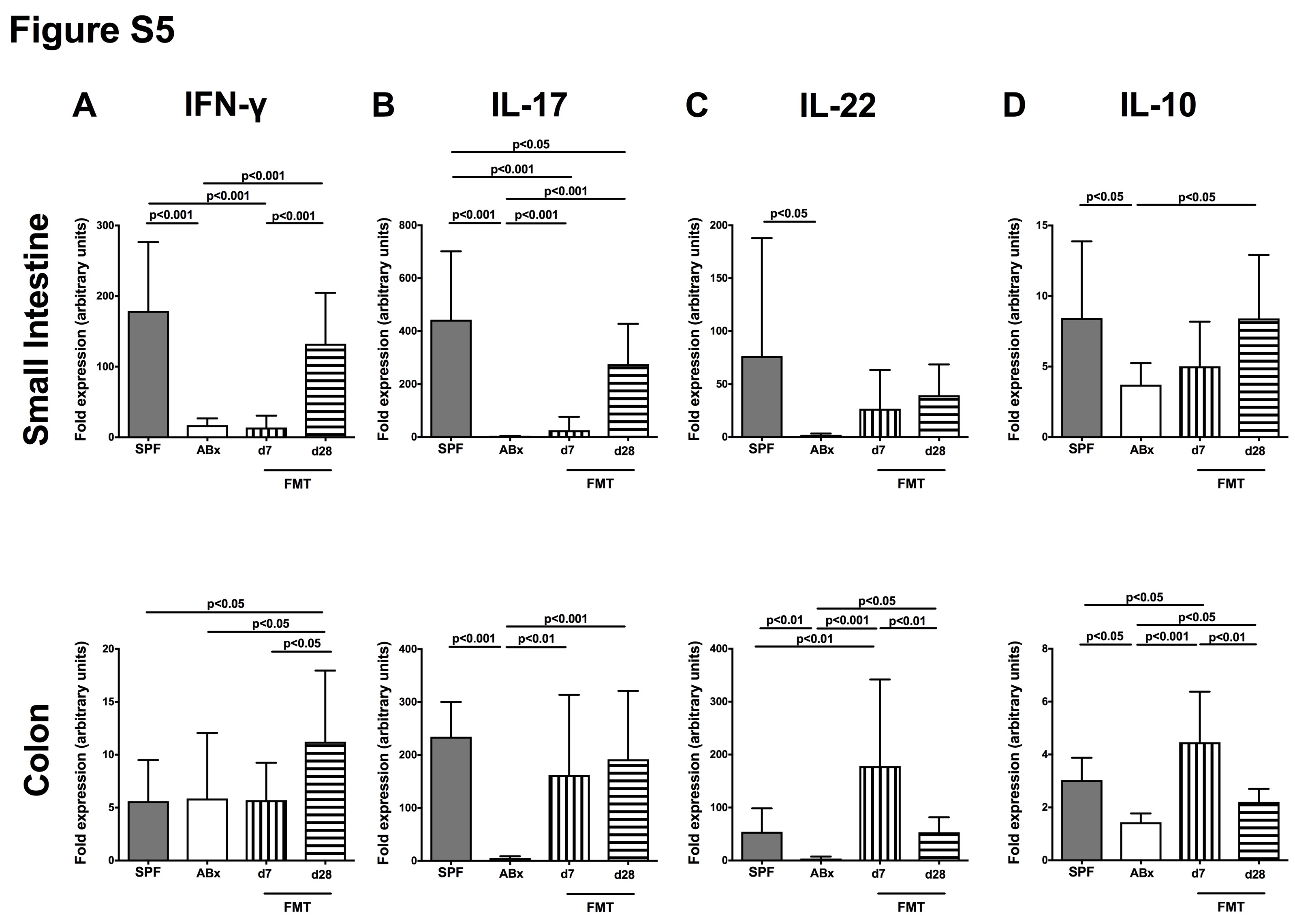

Supplement: Figure S5 — mRNA analysis of pro- and anti-inflammatory cytokines in small intestinal and colonic tissue. RT-PCR results of (A) IFN-γ, (B) IL-17, (C) IL-22, and (D) IL-10 expression in small intestinal (upper panel) and colonic (lower panel) tissues derived from naive conventional mice (SPF, gray bars), secondary abiotic mice (ABx, white bars), and recolonized mice at day (d) 7 (boxes with vertical lines) and d28 (bars with horizontal lines) following fecal microbiota transplantation (FMT) are depicted. [file image_5.jpeg]
